# Protuberances are organized distinct regions of long-term callus: histological and transcriptomic analyses in kiwifruit

**DOI:** 10.1007/s00299-021-02661-0

**Published:** 2021-02-05

**Authors:** Małgorzata Czernicka, Iwona Chłosta, Kinga Kęska, Małgorzata Kozieradzka-Kiszkurno, Mohib Abdullah, Marzena Popielarska-Konieczna

**Affiliations:** 1 Department of Plant Biology and Biotechnology, Faculty of Biotechnology and Horticulture, University of Agriculture, 29-Listopada 54, 31-425 Kraków, Poland; 2 Department of Plant Cytology and Embryology, Faculty of Biology, Institute of Botany, The Jagiellonian University in Kraków, Gronostajowa 9, 30-387 Kraków, Poland; 3 Department of Plant Cytology and Embryology, Faculty of Biology, University of Gdansk, Wita Stwosza 59, 80-308 Gdańsk, Poland

**Keywords:** *Actinidia chinensis* cv. *deliciosa*, Metabolic pathways, Organogenic callus, RNA-seq, Starch granules, Ultrastructure

## Abstract

**Key message:**

Macroscopic, ultrastructural, and molecular features—like a ball shape, the presence of starch granules, and the up-regulation of genes involved in carbohydrate metabolism and secondary metabolite biosynthesis—distinguish PT regions within a callus.

**Abstract:**

The modification of the mass of pluripotent cells into de novo shoot bud regeneration is highly relevant to developmental biology and for agriculture and biotechnology. This study deals with protuberances (PT), structures that appear during the organogenic long-term culturing of callus (OC) in kiwifruit. These ball-shaped regions of callus might be considered the first morphological sign of the subsequent shoot bud development. Sections of PT show the regular arrangement of some cells, especially on the surface, in contrast to the regions of OC beyond the PT. The cells of OC possess chloroplasts; however, starch granules were observed only in PTs’ plastids. Transcriptomic data revealed unique gene expression for each kind of sample: OC, PT, and PT with visible shoot buds (PT–SH). Higher expression of the gene involved in lipid (glycerol-3-phosphate acyltransferase 5 [*GPAT5*]), carbohydrate (granule-bound starch synthase 1 [*GBSS1*]), and secondary metabolite (beta-glucosidase 45 [*BGL45*]) pathways were detected in PT and could be proposed as the markers of these structures. The up-regulation of the regulatory associated protein of TOR (*RAPTOR1*) was found in PT–SH. The highest expression of the actinidain gene in leaves from two-year-old regenerated plants suggests that the synthesis of this protein takes place in fully developed organs. The findings indicate that PT and PT–SH are specific structures within OC but have more features in common with callus tissue than with organs.

**Supplementary Information:**

The online version contains supplementary material available at 10.1007/s00299-021-02661-0.

## Introduction

The formation of plant callus tissue, composed in large part of uniform cells, is not considered a regression in the developmental lineage but rather an opening to increase the developmental potency (Feher 2019). Reprogramming of the callus due to the culture conditions can induce plant regeneration through organogenesis or somatic embryogenesis. Investigation of the development of shoot meristems from a disorganized callus tissue is crucial for basic research and for applications in agriculture and biotechnology (Niazian et al. 2019). Adventitious shoot formation via a callus is a useful method for plant micropropagation and improvement of genotypes through the techniques used in plant biotechnology programs.

One of the most recently domesticated and popular fruit crops is *Actinidia chinensis* var. *deliciosa* A. Chev. (A. Chev.), formerly known as *A*. *deliciosa* (A. Chev.) C. F. Liang et A. R. Ferguson, and commonly called kiwifruit. A wide spectrum of interest in the *Actinidia* genus concern different levels of research—from the taxonomic concept to whole-genome sequencing (Ferguson 2016; Rey et al. 2020).

Previous histological studies revealed that the morphogenic events observed in endosperm-derived callus in kiwifruit led to adventitious shoot bud development (Popielarska et al. 2006; Popielarska-Konieczna et al. 2011). During the experiments each of endosperms isolated from an individual seed gave rise to induction and proliferation of the particular callus line. Further observations indicated that some callus lines maintain a capacity for long-term proliferation and the ability to form shoot buds even after a few years of culturing (unpublished). We suggested that a PT, a ball-shaped region of a callus, is the first morphological sign of shoot bud induction within a kiwifruit callus. The relationship between the appearance of PTs or outgrowths and the emergence of primordia-like structures or shoot buds has been observed during tissue culturing of different species (Nakano and Maeda 1974; Jarret et al. 1981; Fernando et al. 2007; Daffalla et al. 2011; Qiao et al. 2012; Tekdal and Cetiner 2013; Manivannan et al. 2015; Lee and Pijut 2017). However, morpho-histological data about organogenic calluses are focused mainly on structures referred to as protocorm-like bodies (Zhao et al. 2008; Palama et al. 2010; Chen et al. 2019) or nodular structure formation (Oka et al. 1982; Rocha et al. 2012; Corredor-Prado et al. 2015). Histological and ultrastructural studies describing the origin and different stages of in vitro morphogenesis have contributed significantly to the understanding and optimization of regeneration systems (Rocha et al. 2012). These methods are often the first step in recognizing the regions with a presumed ability to form new organs and in undertaking analysis at the molecular level.

Cellular identity, destination, and function are determined largely by the transcriptome, i.e., the complete set of expressed RNA transcripts. Transcriptome profiling is a powerful tool that has been used to estimate the importance of gene products in tissues under different conditions. Moreover, molecular studies could help in distinguishing the structures which are morphologically similar (Chen et al. 2019). Recently, there was increased interest in gene expression during callus induction and proliferation, and then during the regeneration processes from the callus (Xu et al. 2012; Ikeuchi et al. 2013, 2019; Li et al. 2014; Kumari et al. 2017; Nakano et al. 2018; Fu et al. 2019; Gao et al. 2019; Lee and Huang 2019).

The main objectives of the present study were to analyze histological and ultrastructural features and to identify the genes whose expression differed within the OC, especially in PT with and without visible shoot bud meristems. The sections reveal the unique nature of PT and PT–SH. Molecular analysis provides additional evidence to argue that PT are the distinct structures within the OC in kiwifruit. In this research, RNA sequencing allowed for the selection of the genes with more activity in PT than in other regions of the callus. The study on these bulgy structures, which seem to be predicted to form shoot buds, could shed new light on the organization of a callus. As a practical application, e.g., for transformation procedures, protuberances might be considered the proper target, as with protocorm-like bodies (Retheesh and Bhat 2011). To the best of our knowledge, this is the first study to report data which highlights the differences in the expression of selected genes from the transcriptomic analysis and detailed histological and ultrastructural studies of long-term-cultured OC in *Actinidia*.

## Materials and methods

### Plant material and culture conditions

Commercially available fruits of *A*. *chinensis* var. *deliciosa* ‘Hayward’ Zespri^®^ were used as a source of explants. Mature endosperm was isolated from seeds as described previously (Góralski et al. 2005) and cultured under conditions reported by Popielarska-Konieczna et al. (2011). Obtained OC were kept on plates 90 mm diameter and 25 mm height (Stardish, Phoenix Biomedical) and transferred on the fresh medium based on the Murashige and Skoog (1962) (MS) macro-, microelements and vitamins (Duchefa) supplemented with 0.5 mg/l thidiazuron (Sigma Merck) every 4 weeks. The cultures were kept in at the temperature 25/21 °C per day/night and 16-h photoperiod conditions, under cool-white fluorescent tubes (80 µmol photons m^−2^ s^−1^). In the present analyses was used the four years-old callus line no 13, with the proven presence of Y chromosome/-s with using sex-linked molecular markers (data unpublished). The shoots that regenerated (with approx. 1–2 cm of length) were transferred onto a half-strength MS in culture containers, Magenta™ vessels (Sigma), for the root development, which started 2–3 weeks after the sub-culture. Plants were transferred onto fresh medium every 4 weeks, and maintained under temperature and photoperiod conditions as described above. Non-organogenic callus (NOC) induction was obtained and maintained 2 years on MS with the addition of 2 mg/l 2,4-d (Sigma Merck) and 5 mg/l kinetin (Sigma Merck), in the dark.

### Histological and ultrastructural analyses

Pieces of four years old callus with and without PTs were excised from the OC. For histological analyses, the samples were fixed in 5% (w/v) glutaraldehyde (GLA) in 0.1 M PBS (pH 7.2). Embedding in synthetic resin Technovit 7100, cutting, staining with 1% (w/v) toluidine blue (TBO). Some sections were stained with 0.01% (w/v) auramine O, and immediately observed by a fluorescence microscopy (Nikon Eclipse E400) with the Nikon filter sets B2-A. Periodic acid-Schiff (PAS) reaction procedure was conducted for the visualization of insoluble polysaccharides (e.g. starch and cellulose). The sections were treated with 0.5% (w/v) periodic acid for 10 min and rinsed in distilled water. Sections were then stained with Schiff’s reagent for 30 min, and rinsed in rinsing solution. Some sections were stained with a saturated solution of Sudan III in 92% ethanol (w/v) for 2 h at 37 °C, washed and mounted in glycerol. Slides after TBO, PAS and Sudan III treatments were observed with bright-field illumination using a microscope Nikon Eclipse E400.

For the scanning electron microscopy (SEM) analyses, the callus samples were prefixed in 5% (w/v) GLA in 0.1 M PBS (pH 7.2), dehydrated in a graded ethanol series, dried with a CO_2_ critical point drying (CPD) system, sputter-coated with gold and observed with a HITACHI S-4700 electron microscope.

For the transmission electron microscopy (TEM) analyses, the samples of callus were prefixed in 2.5% (w/v) GLA, 2.5% (w/v) formaldehyde in 0.1 M cacodylate buffer (pH 7.0), respectively. The samples were post-fixed in buffered 1% (w/v) OsO_4_ overnight. The samples were treated with 1% (w/v) uranyl acetate, dehydrated in a graded acetone series, and then embedded in Spurr’s resin. Sections were obtained with using a Sorvall MT-2B microtome, stained with uranyl acetate and lead citrate, and examined with a Philips CM 100 electron microscope.

### RNA extraction

Samples of NOC and OC were frozen in liquid nitrogen and stored at − 80 °C. Samples of only clear visible ball-shaped PTs were excised from the OC using a stereoscopic dissecting microscope. PT and PT–SH were collected separately, and adventitious shoots were discarded. In the analysis, leaves from regenerated plants at three developmental stages were included: (i) at early/young stage—4–6 weeks after the transfer onto a half-strength MS (REG-Y), (ii) at a medium stage—6–8 months after the transfer onto a half-strength MS (REG-M), (iii) at an elder stage—two years after the transfer onto a half-strength MS (REG-E). Plants from all stages were maintained under in vitro conditions.

The total RNA extraction was carried out with the Nucleospin RNA Plant and Fungi kit (Macherey–Nagel) following the manufacturer’s instruction. RNA samples were treated with 1 U/µl RNase-free DNase I (Ambion Inc.) and 40 U/µl RiboLock RNase Inhibitor (Thermo Fisher Scientific) to avoid DNA contamination and RNA degradation. The quality and integrity of RNA samples were verified by electrophoresis in 1% (w/v) agarose gel in denaturing conditions. RNA concentration and quality were evaluated spectrophotometrically using NanoDrop 2000c (Thermo Scientific™) by the absorbance at 230, 260, and 280 nm wavelengths measurements.

### RNA-seq library construction and Illumina HiSeq4000 sequencing

From RNA samples, mRNA was obtained using NEBNext^®^ Poly(A) mRNA Magnetic Isolation Module (NEB). High quantity RNA samples (OD260/280 = 2.0–2.2, OD260/230 ≥ 2.0, RIN ≥ 8.0, 28S:18S ≥ 1.5), assessed using Agilent Bioanalyzer 2100 (Agilent Technologies), were employed to construct the sequencing library using NEBNext^®^ Ultra™ Directional RNA Library Prep Kit for Illumina (Illumina). High-throughput sequencing of cDNA in PE100 (paired ends mode, 100 bp) was performed using Illumina HiSeq4000 platform. Each experiment included three biological replicates. The RNAseq datasets generated for this study are deposited in the NCBI under BioProject PRJNA669335 and BioSample under Acc. No. SAMN16456073, SAMN16456074, SAMN16456075, SAMN16456076, SAMN16456104, SAMN16456106, SAMN16456107, SAMN16456108, SAMN16456113, SAMN16456114, SAMN16456116, SAMN16456117 and can be found in the GenBank Short Read Archive (SRA) under Acc. No. from SRR12881583 to SRR12881594.

### Bioinformatic analysis of RNAseq data

Raw sequencing data, in FASTQ format were filtered using Trimmomatic (Bolger et al. 2014) to remove TRueSeq3-PE adapters, reads with > 10% unknown nucleotides (N) bases, low quality (*Q* value < 20) and < 30 bp in lenght reads. The quality control was performed again using FastQC ver. 0.11.3 (Andrew 2010). Clean reads from NOC, OC, PT and PT–SH samples were employed for de novo assembly using Trinity ver. 2.4.0) (http://trinityrnaseq.github.io) (Grabherr et al. 2011). The contigs were clustered and further assembled according to paired-end information and the resulting sequences were defined as unigenes. Each unigene represented a collection of expressed sequences i.e. transcripts. This set of unigenes and transcripts were kept for downstream analyses. All assembled unigenes were annotated using the Trinotate pipeline ver. 3.1.0 (https://trinotate.github.io). Protein-coding regions in the unigenes were predicted based on the most likely longest-ORF using TransDecoder-v3 (Haas et al. 2013). Homolog sequences for the predicted proteins were searched using BLASTP and BLASTX with *E*-value ≤ 1E−10 against the SwissProt/Uni-Prot database. In both cases, BLASTP and BLASTX, we only kept top-hit matches. Protein domains were identified using HMMER-3.1b2 (Finn et al. 2011) to the Pfam database (ftp://ftp.ebi.ac.uk/pub/databases/Pfam/). Homologous proteins found in the SwissProt database were used to retrieve functional annotation comments from the GO (*Gene Ontology)* (Ashburner et al. 2000), EggNOG (*Evolutionary Genealogy of Genes: Non-supervised Orthologous Groups)* (Powell et al. 2012). Metabolic pathway analysis of the assembled transcripts was performed according to the KEGG (*Kyoto Encyclopedia of Genes and Genomes)* database (Kanehisa et al. 2012). Unigene and transcript abundances for each library was calculated by RSEM ver. 1.3.0 http://deweylab.github.io/RSEM/package) (Li and Dewey 2011). The FPKM (fragments per kilobase of exon per million mapped fragments) method was used to determine the expression level of each transcript. Differentially expressed genes (DEGs) among each libraries were calculated by using the Empirical Analysis of Digital Gene Expression—edgeR ver. 3.20.1) statistical package (Robinson et al. 2010). The trimmed mean of M-values (TMM) method was used to calculate the normalization factors. The false discovery rate (FDR) < 0.05 was used as criteria for identifying significant differences in expression. The main goal was the comparison of the expression patterns between NOC and OC, and following developmental stages of OC (OC, PT and PT–SH) and for that reason we identified DEGs between OC versus NOC, PT versus OC and PT–SH versus PT. Additionally, we performed comparisons between PT versus NOC and PT–SH versus NOC and also PT–SH versus OC, because they were helpful in selection exclusively and common expressed DEGs for each samples. GO enrichment analysis conducted on each comparison independently was performed using topGO in R ver. 2.34.0. (Alexa and Rahnenfuhrer 2018) with Fisher exact test considering FDR ≤ 0.001. KEGG pathway (https://www.kegg.jp/kegg/pathway.html) enrichment of DEGs were performed using clusterProfiler ver. 3.16.1) (Yu et al. 2012). The DEGs were considered to be significantly enriched when a Bonferroni-adjusted *p *value ≤ 0.05 was obtained.

### qRT-PCR analysis of gene expression

For expression analysis, seven genes involved in carbohydrate and lipid metabolism, cell wall modification, epidermis development, the synthesis of peptidase and cell expansion were chosen and based on RNA-seq analysis they were identified as differentially expressed in the OC, PT and PT–SH (Table S1). For a design of gene-specific primers, transcript sequences de novo assembled from RNA-seq were used (sequences have been deposited in GenBank, Acc No. MT451975, MT451977, MT451978, MT451979, MT460908, MT463287). Additionally, to determine genomic sequences of genes, each transcript was used as query sequence to blast the Kiwifruit Genome Database (Huang et al. 2013). All the primer sequences used in this study were listed in Table S1. 1 ug of each RNA probe was transcribed into cDNA using an iScript cDNA synthesis kit (BioRad), following the manufacturer's instruction. cDNA synthesis was conducted in three biological replicates. The obtained cDNA was frozen at − 20 °C until use. The qPCR analysis was performed with a StepOnePlus™ Real-Time PCR System (Applied Biosystems). The reaction mixture included: 12.5 µL Maxima™ SYBR Green/ROX qPCR Master Mix (2X) (Thermo Scientific), 0.7 µL of (5 µM) each primer (forward and reverse), 2 µL fivefold diluted template cDNA and in a total volume of 25 µL made up with nuclease-free DEPC-treated water (diethylpyrocarbonate; Thermo Fisher Scientific). Finally, highly specific primers for expression experiments were designed using Primer3Plus (https://primer3plus.com/cgi-bin/dev/primer3plus.cgi) based on the genomic and transcript sequences (Table S1). The absence of primer-dimer and hairpin structures was determined by the IDT-OligoAnalyzer ver. 3.1 program (https://eu.idtdna.com/calc/analyzer). The utility of designed primers were validated using RT-PCR reaction and confirmed by electrophoresis in 1% (w/v) agarose gel and primers specificity were verified analyzing one single peak in all melting curves. The qPCR method described above was linked to the MIQE guidelines (Bustin et al. 2009). The qPCR cycling conditions were as follows: denaturation 95 °C/10 min; 40 cycles at 95 °C/15 s, 60 °C/30 s, 72 °C/30 s. The melting curves were obtained by melting the amplified template from 60 °C to 95 °C/15 s increasing the temperature by 0.3 °C per cycle. Each qPCR reaction was conducted in three technical replicates among three biological ones. Each plate also incorporated a no-template control. Amplification efficiencies for all primer pairs were evaluated using serial tenfold dilutions of pooled cDNA. The efficiency of each qPCR was calculated from the slope of the standard curve using the formula *E* = 10^−1/slope^ and converted into percentage values according to the following formula %*E* = (*E* − 1) × 100%. Actin (*act1*) and 18S ribosomal RNA (*18S*) were considered as endogenous reference genes (Table S1). Relative quantification of gene expression was calculated using the $2^{{ - \Delta \Delta C_{{\text{t}}} }}$ method (Livak and Schmittgen 2001). Fisher’s exact test was applied to determine statistical differences between tested developmental stages (*p* < 0.05).

## Results

### Morphological, histological and ultrastructural characterization of OC, PT, and PT–SH

**Fig. 1:**
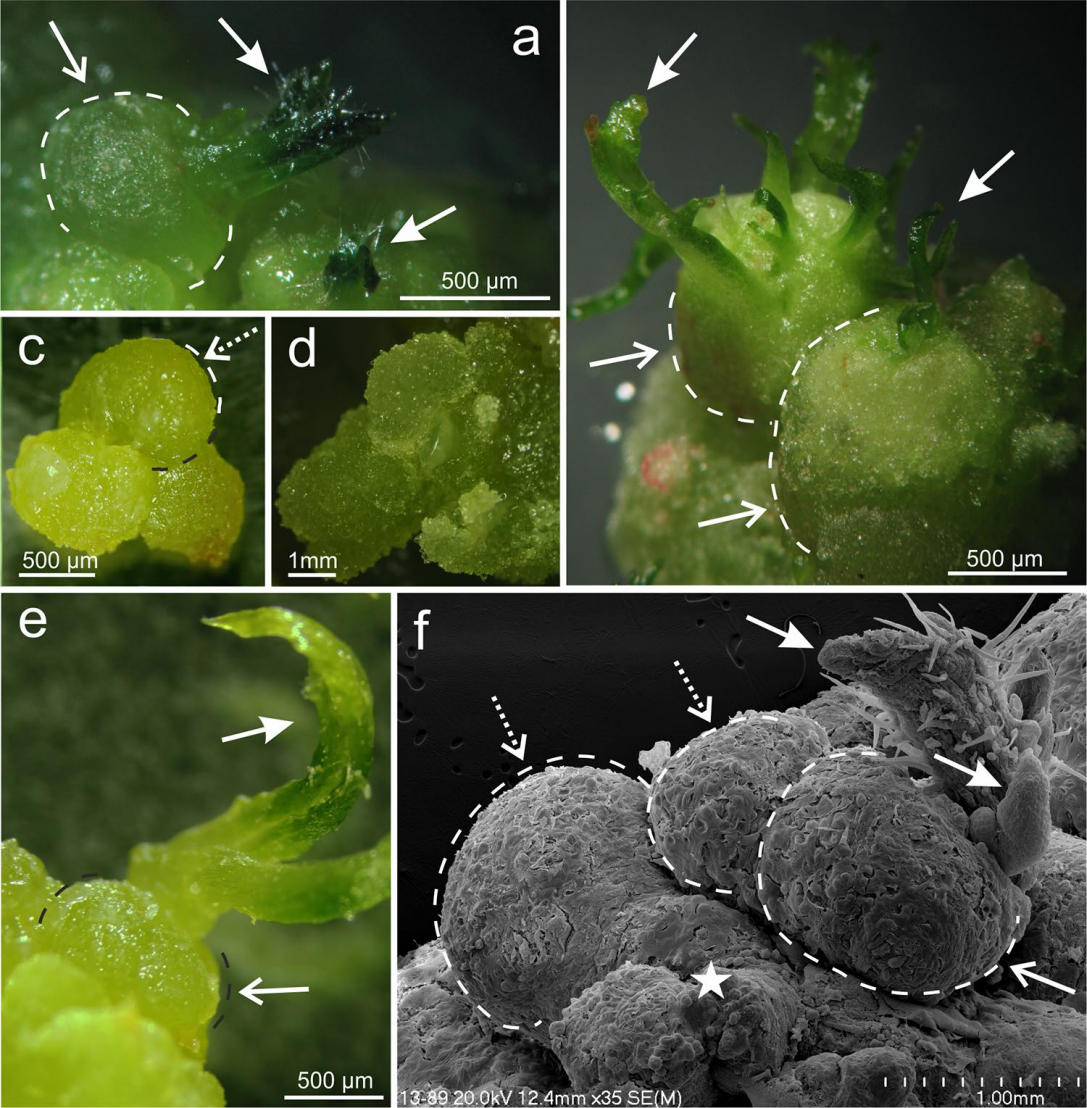
Morphology (**a**–**e**) and SEM images (**f**) of OC and PT in *Actinidia chinensis* cv. *deliciosa*. **a**, **b** Ball-shaped PTs (open arrows) with shoot buds (arrows) at early (**a**) and subsequent (**b**) stages of the growth of shoots. **c** The detached PT without shoot buds. **d** Structure of the callus beyond PT. **e** PT with developing adventitious shoots. **f** Callus formed only PT (dotted arrows) and PT (open arrows) with shoot buds (arrows); asterisks indicate regions of the callus without the structure and shape typical of PT. *Lineolate lines* highlight the ball shape of PTs (**a–e**)

The 4-year-old callus from line no. 13 had an intense green color and firm structure (Fig. 1a–e). Through the whole culture, there has been a continued organogenic ability to produce new adventitious shoot buds. Shoot buds arose from previously formed PTs, ball-shaped structures (Fig. 1a–c, e), which could be clearly distinguished from other regions of the callus (Fig. 1d). PT and PT–SH could be easily detached from the explant (Fig. 1c). SEM observations revealed that the PT are covered with more or less closely attached cells and a membranous layer (Fig. 1f). Globular cells of different sizes were observed on the surface of the shapeless part of the OC beyond the PT (Fig. 1f).

**Fig. 2:**
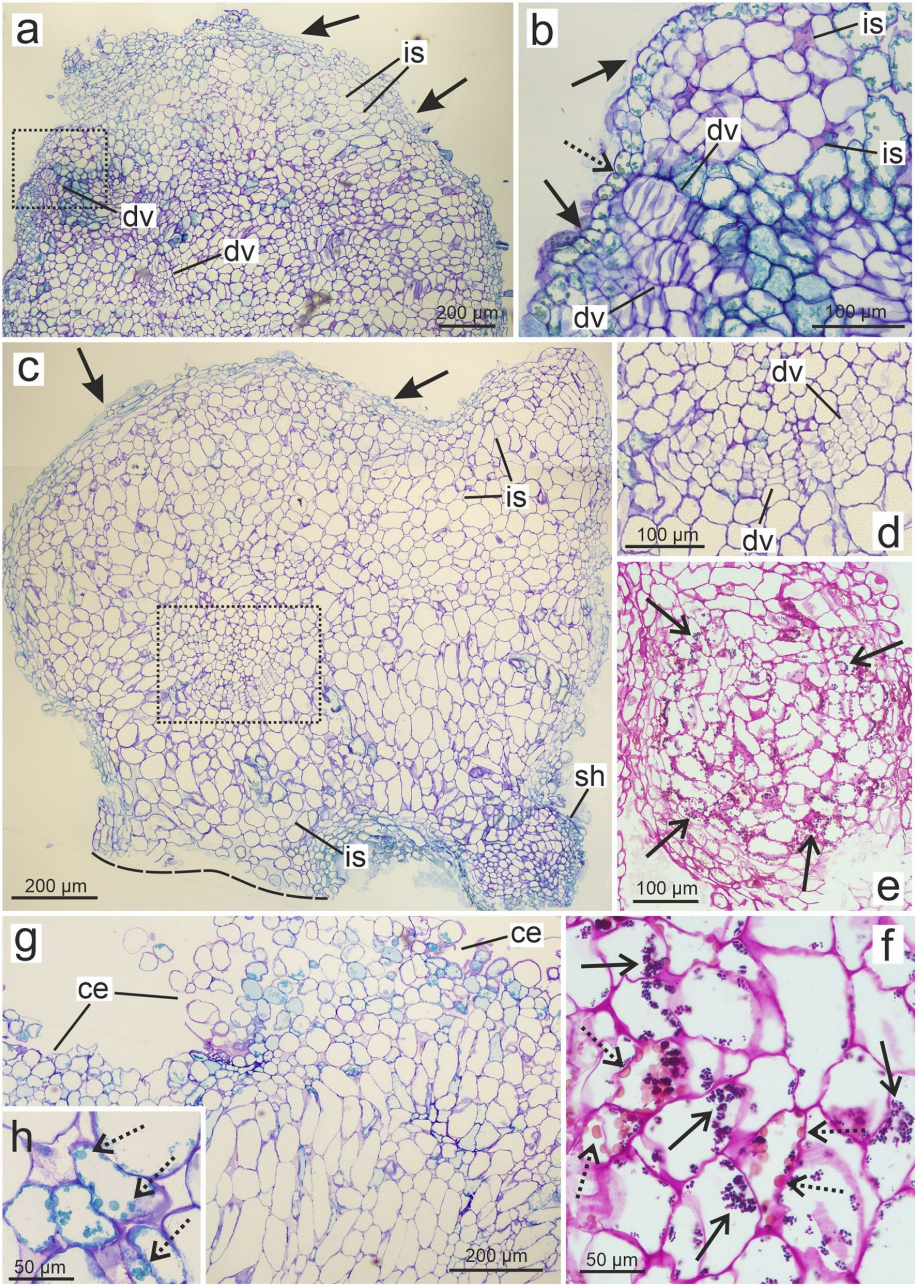
Longitudinal sections of detached PTs (**a**–**f**) and OC (**g**, **h**) in *Actinidia chinensis* cv. *deliciosa*; TBO (**a**–**d**, **g**, **h**) and PAS (**e, f**) staining. **a** Bulgy PT with visible coordinated divisions (*dv*) and compact composition of cells (arrows) on the surface. **b** Magnification of the *rectangle* from **a** shows cells originated from coordinated divisions (*dv*) and cells with regular composition (arrows) on the surface; the deposition of phenols stained greenish (dotted arrows). **c** Ball-shaped PT with region of cells formed through coordinated divisions (*dv*); visible regular, composed cells on the surface (arrows); *lineolate line* indicates the site of dissection from the rest of the callus. **d** Magnification of the *rectangle* from **c** shows cells clusters formed after coordinated divisions (*dv*). **e** Part of the PT composed with the cells, which possess an abundance of blue-violet stained starch grains (open arrows). **f** Magnification of cells with the starch grains (open arrows) and the deposition of phenols stained orange (dotted arrows). **g** The surface composed of loosely attached cells (*ce*) was characteristic of the part of the OC beyond PT. **h** Phenolic deposits (dotted arrows) inside the cells. Arrows regular composition of cells on the surface; *dv* cells derived from coordinated divisions; dotted arrows phenolic deposits; open arrows starch grains; *is* intercellular spaces; *sh* adventitious shoot bud

Histological analysis of sections of the bulgy and ball-shaped structures revealed that some cells on the surface and inside of these PT are arranged in a regular manner (Fig. 2a–d). The intercellular spaces were also clearly visible inside and near the surface of the PT. The clusters of cells originated from coordinated divisions were regularly arranged and closely attached (Fig. 2a–d). Numerous starch grains in cells located near the surface of the PT were observed (Fig. 2e, f). Phenolic deposits were observed in many cells as greenish (after TBO staining; Fig. 2b) or orange (after PAS reaction; Fig. 2f) vacuolar inclusions. The samples taken from the OC beyond PT were composed of loosely attached cells, and their appearance was irregular, especially on the surface (Fig. 2g). Some cells contained phenolic deposits (Fig. 2h). After the application of auramine O, the fluorescence of the cell wall was detected in the cells which composed the surface of the PT (Suppl. Figure 1a–c). Weak autofluorescence of the cell wall in cells located inside of the PT was observed. A similar weak fluorescence was found for all cells of the OC beyond PT (Suppl. Figure 1d, e). Staining with Sudan III revealed a reddish color in the outer cell layer of PT (Suppl. Figure 2), which corresponds with fluorescence after the application of auramine O.

**Fig. 3:**
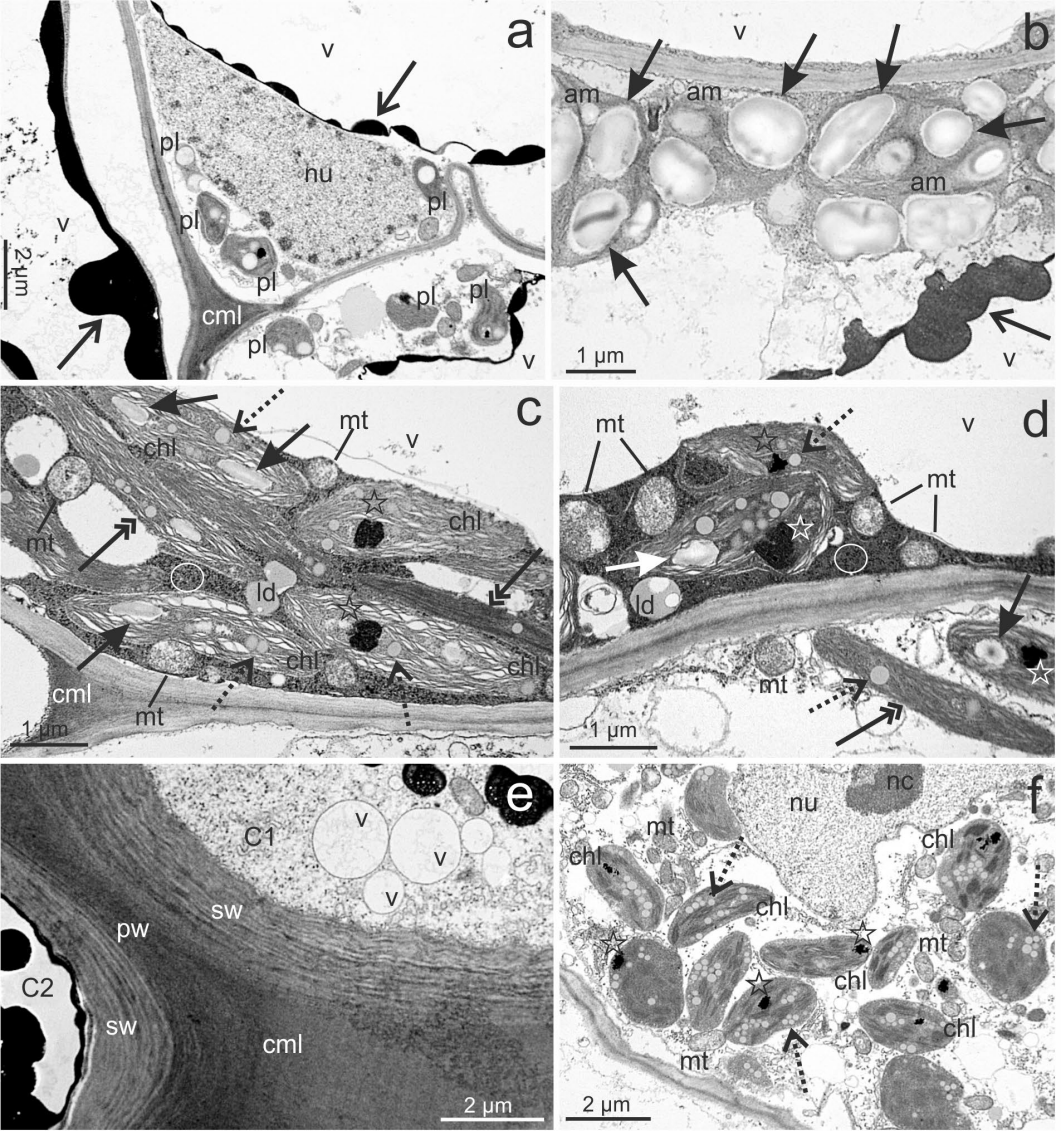
TEM images of cells, which form PT (**a**–**e**) and OC (**f**) in *Actinidia chinensis* cv. *deliciosa*. **a** Cells with an abundance of the deposition of phenols (open arrows) in vacuoles (*v*), laterally located nucleus (*nu*) and plastids (*pl*). **b** Amyloplasts (*am*) with numerous starch grains (arrows) and deposition of phenols (open arrows) in vacuole (*v*). **c**, **d** Chloroplasts (*chl*) with starch grains (arrows), plastoglobules (dotted arrows) and an electron-dense inclusions (stars); visible mitochondria (*mt*), lipid droplets (*ld*), dense cytoplasm with ribosomes (circles); note elongated chloroplasts (double arrows). **e** Cells from the surface of PT with thick cell wall composed of the primary cell wall (*pw*) and lamellate structure of the secondary cell wall (*sw*); note cell middle layer (*cml*) between cells (*C1*, *C2*).** f** Chloroplasts (*chl*) with plastoglobules (dotted arrows) and an electron-dense inclusions (stars); visible part of lobed nucleus (*nu)* with nucleolus (*nc*)

The cells within PT were highly vacuolated with a large number of phenolic deposits (Fig. 3a). The nucleus, plastids, and other organelles were located peripherally, near the plasmalemma and the cell wall. Amyloplasts with large starch grains were observed (Fig. 3b). Chloroplasts contain thylakoids, plastoglobules, electron-dense inclusions, and starch grains (Fig. 3c, d). Some chloroplasts demonstrated an elongated shape. In PT cells, the mitochondria, lipid droplets, phenolic deposits, and numerous ribosomes were visible (Fig. 3c, d). Cells located near the surface of PT had a thick cell wall composed of the primary cell wall and the lamellate structure of the second cell wall (Fig. 3e). The cell middle lamella was partially highly extended. Cells of the callus beyond PT contained the mitochondria and chloroplasts without starch grains; however, no lipid droplets were found (Fig. 3f).

### Comprehensive transcriptome analysis of OC, PT, and PT–SH

RNA sequencing of three biological replicates from the NOC, OC, PT, and PT–SH, a total of 24 samples, was performed on the Illumina HiSeq 4000 platform. The highest number of clean reads were generated in the OC samples (in total 99,694,899 clean reads and 9.96 Gbp, with an average of 3.32 Gbp per sample) (Table S2). For the PT samples, 15% fewer clean reads were obtained (in total 84,652,609 clean reads and 8.46 Gbp, with an average of 2.82 Gbp per sample). The average GC content was approximately 47% in the samples and the clean reads were of high quality (Q39 bases) (Table S2).

The clean reads from the all samples were subjected to assembly, resulting in 652,203 transcripts with an N50 of 944 bp. Among them, the longest transcript was 15,057 bp, and the average transcript length was 697 bp (Table S3). After identifying isoforms (different transcripts of a single unigene), a total of 260,372 unigenes with an N50 of 620 bp were generated. The average length of the unigenes was 514 bp, ranging from 201 to 14,092 bp (Table S3). Most of the unigenes (about 90%) were < 1000 bp in length.

**Fig. 4:**
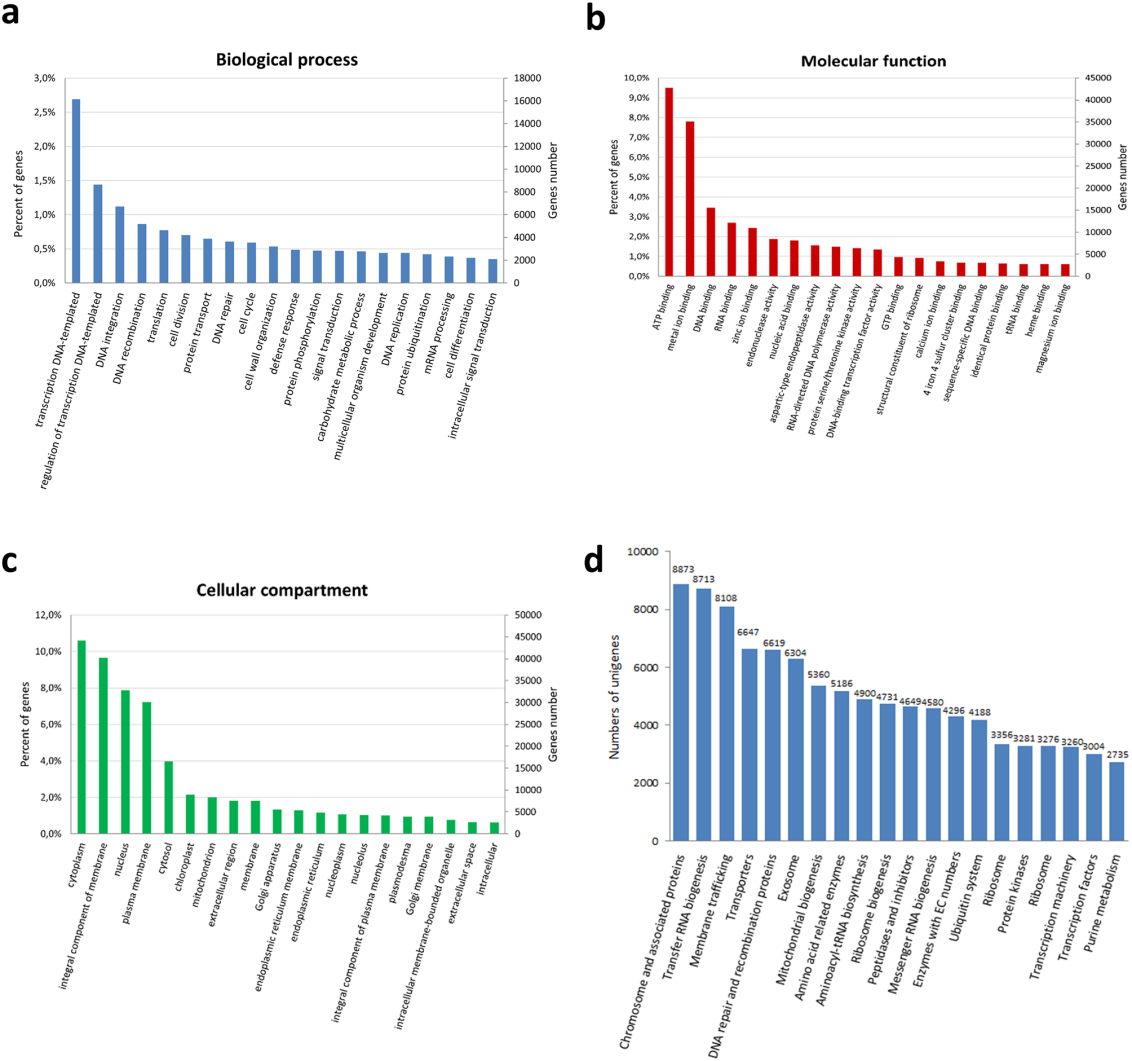
Functional annotations of unigenes based on Gene Ontology (GO) system, i.e., dominant subcategories of biological process (**a**), molecular function (**b**) and cellular compartment (**c**) categories and the top 20 Kyoto Encyclopedia of Genes and Genomes (KEGG) pathways containing the most unigenes (**d**)

To identify the putative function of the assembled unigenes of *A. chinensis* var. *deliciosa*, they were searched against the SwissProt/Uni-Prot, Pfam, EggNOG, GO, and KEGG databases using the BLASTP and BLASTX algorithms, with an *E*-value of less than 1.0 × 10^–10^. In total, 225,748 (86.7%) unigenes were found in at least one of these databases, of which 221,350 unigenes were aligned successfully to known proteins via BLASTX; BLASTP exhibited significant hits for 70,015 unigenes. Among these unigenes, 28,621 and 158,562 sequences matched successfully to records in the Pfam and eggNOG databases, respectively. The functions of the *A. chinensis* var. *deliciosa* unigenes were classified via GO analysis. In total, 215,195 unigenes were successfully categorized into 23,477 functional groups and they were classified into three major GO categories—biological processes, molecular function, and cell component—which were represented by 14,823, 2300, and 6354 GO terms, respectively (Suppl. data S1). The dominant subcategories of the classified genes in the category of biological processes included transcription DNA-templated (16,196), DNA integration (6729), DNA recombination (5181), translation (4646), cell division (4198), protein transport (3893), DNA repair (3626), cell cycle (3538), cell wall organization (3197), protein phosphorylation (2854), signal transduction (2827), carbohydrate metabolic process (2784), and multicellular organism development (2639) (Fig. 4a); in the molecular function category were ATP binding (42,842), metal ion binding (35,141), DNA binding (15,569), RNA binding (12,155), zinc ion binding (10,958), endonuclease activity (8471), protein serine/threonine kinase activity (6355), DNA-binding transcription factor activity (6099), and GTP binding (4371) (Fig. 4b); the cell component category included cytoplasm (44,239), integral component of membrane (40,217), nucleus (32,780), cytosol (16,532), chloroplast (8898), mitochondrion (8339), extracellular region (7609), Golgi apparatus (5557), endoplasmic reticulum membrane (5404), plasmodesma (3987), extracellular space (2710), and cell wall (2114) (Fig. 4c).

The KEGG pathway analysis provided annotations of 186,284 unigenes that were distributed to 519 KEGG pathways (Suppl. data S1). The most unigene sequences (59,389) exhibited matches to *Arabidopsis thaliana* pathways. Among the annotated pathways, the “chromosome and associated proteins,” “transfer RNA biogenesis,” “membrane trafficking,” “transporters,” “DNA repair and recombination proteins,” “amino acid related enzymes,” “mitochondrial biogenesis,” and “peptidases and inhibitors” pathways were the most abundant (Fig. 4d).

Based on the de novo assembled transcriptome as a reference, the genes expressed in the NOCs and three kinds of OC (OC, PT, and PT–SH) were identified. Differential expression analysis was based on fragments per kilobase of transcript per million (FPKM) and an FDR < 0.05. The DEG numbers are shown in Table S4.

**Fig. 5:**
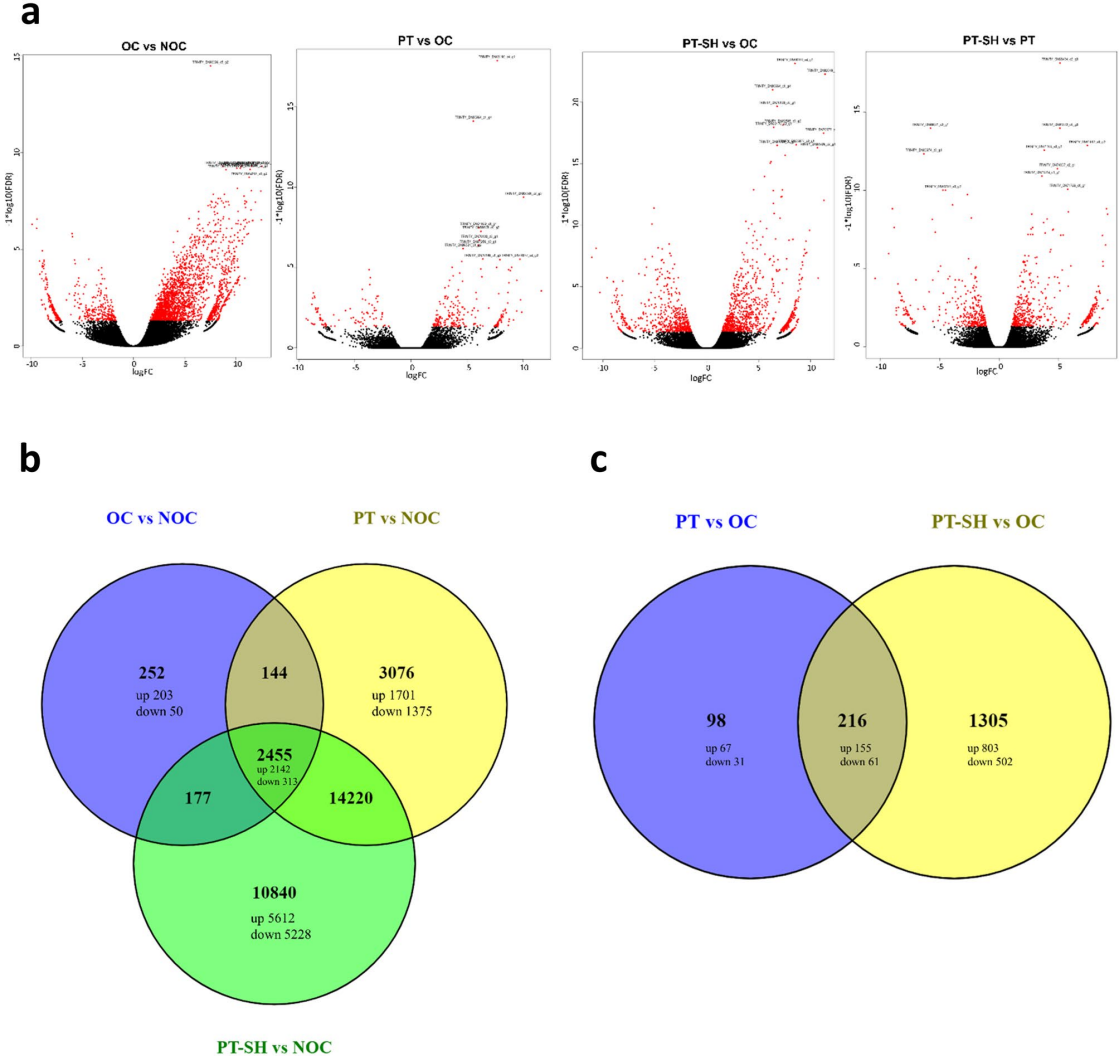
Differentially expressed genes (DEGs) in OCs, PTs, and PT–SH presented in the volcano plots for OC vs. NOC, PT vs. OC, PT–SH vs. OC, and PT–SH vs. PT comparisons; the red dots represent DEGs (FDR < 0.05) in each comparison (**a**) and in Venn diagrams for OC vs. NOC, PT vs. NOC, and PT–SH vs. NOC (**b**) and PT vs. OC and PT–SH vs. OC comparisons (**c**)

Compared with the NOC, 3028 significant DEGs were identified in the OC; in the PT and PT–SH, the number of DEGs was 6.5 and 9.1 times higher, respectively. Among the DEGs detected in OC, the number of up-regulated genes was 2584 and there were 6 times fewer (444) down-regulated genes (Table S4 and Fig. 5a). We compared the three data sets from comparisons versus the NOC group (Fig. 5b). The Venn diagram analysis of DEGs revealed a total of 2455 commonly expressed genes in the OC, PT, and PT–SH and 252 genes exclusively expressed in OC, of which 203 were up-regulated.

Differential gene expression analysis among the three kinds of OC revealed 314 DEGs between PT and OC, 1521 DEGs between PT–SH and OC, and 885 DEGs between PT–SH and PT (Table S4). Among them, up-regulated genes were predominant, though the number of such genes is 0.6 times higher for PT vs. OC combination, 0.4 times for PT–SH vs. OC, and only 0.3 times higher for PT–SH vs. PT (Fig. 5a). The Venn diagram (Fig. 5c) revealed 216 commonly expressed DEGs in the PTs and PT–SH compared with the OC, and 98 and 1305 DEGs exclusively expressed in PTs and PT–SH. Detailed characteristics of common and specific DEGs for each combination are enclosed as Suppl. data S2.

To obtain insight into the functional categories of the DEGs induced in the transformation of the OC, a GO enrichment analysis was performed using TopGO (Fisher’s exact test; FDR ≤ 0.001). The GO enrichment analysis showed that the most significant GO DEGs in the biological process (BP) category were assigned to PT–SH vs. PT, and most DEGs were assigned to the molecular function (MF) and cellular component (CC) categories in the OC vs. NOC combination (Table S5). Among all combinations, the number of up-regulated DEGs was greater than the down-regulated ones. A total of 1851, 462, and 7778 DEGs were annotated by the BP terms derived from OC vs. NOC, PT vs. OC, and PT–SH vs. PT comparisons, respectively. For the biological process classification in the OC vs. NOC combination, the most significant enriched GO categories were photosynthesis, followed by oxylipin biosynthetic process, secondary metabolite biosynthetic process, oxidation–reduction process, lignin biosynthetic process, plant-type primary cell wall biogenesis, and response to oxidative stress (Table S6; Suppl. data S3). In the PT vs. OC comparison, the regulation of biological process showed 375 (81.2%) up-regulated genes (Table S5) and among all DEGs the most significant enriched GO categories sequentially were carbohydrate metabolic process, followed by plant-type primary cell wall biogenesis, cell wall organization, basic amino acid transport, multidimensional cell growth, and finally auxin catabolic process (Table S6; Suppl. data S3). In the aspect of enrichment of DEGs for the PT–SH vs. PT combination in the biological process category, the most significant enriched GO categories were DNA-templated transcription, water transport, response to abscisic acid, xyloglucan metabolic process, auxin catabolic process, hydrogen peroxide catabolic process, and urea transport (Table S6; Suppl. data S3).

The next stage was to select uniquely and commonly enriched GO terms in the BP category associated with each kind of OC. The Venn diagrams showed an overlap of BP GO terms relative to NOC among OC, PT, and PT–SH (Suppl. data S3:file 4). Thanks to this approach, in the OC, 26 uniquely enriched GO categories were indicated, involved in photosynthetic electron transport, stabilization and assembly of PSII, negative regulation of brassinosteroid biosynthetic process, positive regulation of development, somatic embryogenesis, regulation of cutin biosynthetic process, lignan biosynthetic process, 1-deoxy-d-xylulose 5-phosphate biosynthetic process, and stem vascular tissue pattern formation, among others. Venn diagrams showing an overlap of BP GO terms among PT and PT–SH relative to OC (Suppl. data S3) led to the identification of 12 unique GO terms for PT, i.e., beta-glucan biosynthetic and metabolic process, carbohydrate biosynthetic process, starch biosynthetic process, mitotic cytokinesis, and multidimensional cell growth and 70 specific BP GO categories for PT–SH, including response to abscisic acid and lipids, transport of water, urea, phosphate ion, hemicellulose metabolic process, suberin biosynthetic process, metabolic process of l-asparagine, phenylpropanoid, and brassinosteroid.

In the MF category classification, the most highly enriched MF terms by DEGs derived from the OC vs. NOC combination were binding and catalytic activity (Table S6; Suppl. data S4). In the binding subset, binding of pigment, iron ion, chlorophyll, and DNA transcription factor activity were significantly higher than the others. In catalytic activity, the most significant terms represented oxidoreductase activity, i.e., glyceraldehyde-3-phosphate dehydrogenase activity and linoleate 13S-lipoxygenase activity. In the PT vs. OC combination, the number of DEGs assigned to catalytic activity and binding was significantly higher than the other classifications (Table S6; Suppl. data S4). In the first subset were the activities of beta-galactosidase activity, cellulose synthase, and xyloglucan 1,6-alpha-xylosidase, and the binding MF category was represented by carbohydrate binding. For the PT–SH vs. OC combination, enrichment of DEGs in the MF category resulted in assignment to five subcategories: catalytic activity, transcription regulator activity, structural molecule activity, transporter activity, and binding (Table S6; Suppl. data S4:file 3). Venn diagrams presenting an overlap of MF GO terms among OC, PT, and PT–SH relative to NOC (Suppl. data S4) showed 29 unique GO terms for OC including the activity of: acylglycerol lipase, long-chain-(S)-2-hydroxy-long-chain-acid oxidase, carbohydrate transmembrane transporter, and 1-deoxy-d-xylulose-5-phosphate synthase. Venn diagrams for combinations of PT and PT–SH relative to OC (Suppl. data S4) indicted three unique GO terms for PT, i.e., glycerate dehydrogenase, xyloglucan 1,6-alpha-xylosidase, and alpha-d-xyloside xylohydrolase activity, and 59 unique MF GO categories for PT–SH, including activity of water transmembrane transporter, DNA binding transcription factor, xyloglucan:xyloglucosyl transferase, symporter, and asparagine synthase (glutamine-hydrolyzing).

In the CC classification, 42 GO terms were assigned to DEGs derived from the OC vs. NOC combination and 8 and 12 GO terms for DEGs from the PT vs. OC and PT–SH comparisons, respectively (Table S5). The most numerous group of downregulated genes was for PT–SH vs. PT (Table S4). Under the cellular component category, in the OC vs. NOC comparison most DEGs were assigned to chloroplast, PSI and PSII, integral component of membrane, and apoplast (Table S5; Suppl. data S5). In the PT vs. OC comparison, the highest number of DEGs were correlated with apoplast, cell wall, anchored component of membrane, and complex of cellulose synthase (Table S5; Suppl. data S5). In the PT–SH vs. PT combination, the most down-regulated DEGs were related with vacuole, primary cell wall, apoplast, and integral component of plasma membrane (Table S5; Suppl. data S5). Venn diagrams showing an overlap of CC terms among OC, PT, and PT–SH relative to NOC (Suppl. data S5:file 4) identified 16 unique GO terms for OC associated mainly with chloroplast and complexes of oxidoreductase and NAD(P)H dehydrogenase. Venn diagrams for combinations of PT and PT–SH relative to OC (Suppl. data S5:file 5) identified two unique CC GO terms for PT: anchored component of membrane and cellulose synthase complex. For the PT–SH vs. OC combination, enrichment of DEGs in the CC category resulted in assignment to 13 subcategories related with vacuole, plasma membrane, photosystem I, and etioplast (Suppl. data S3).

**Fig. 6:**
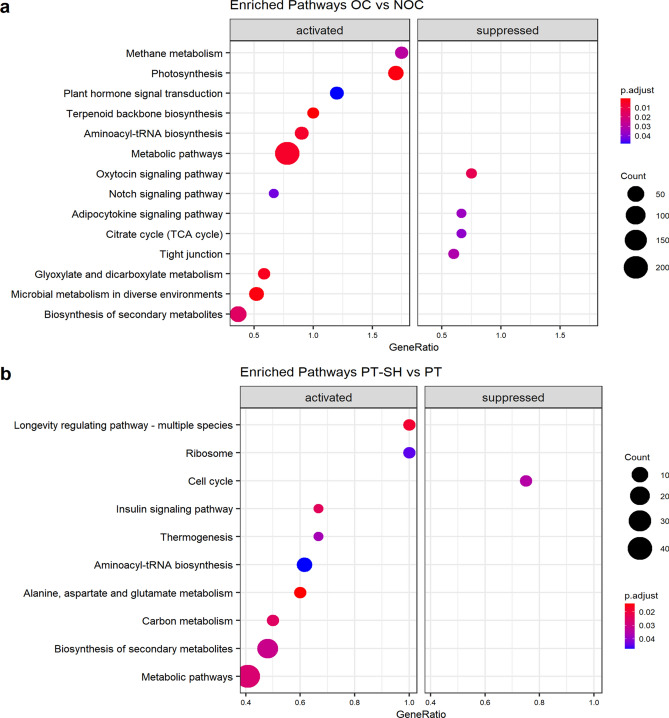
Pathway enrichment of DEGs based on KEGG during the transformation from OC to PT–SH for comparisons, i.e., OC vs. NOC (**a**) and PT–SH vs. PT (**b**); the area of bubbles indicates the number of enriched DEGs, the color of the bubbles indicates *p* adjusted value; up- and down-regulated DEGs are defined as activated and suppressed, respectively

The pathways that displayed significant changes during transformation from OC to PT–SH were identified using the KEGG database. A total of 21 KEGG pathways were significantly enriched only for OC vs. NOC and PT–SH vs. PT combinations (Fig. 6), among which “biosynthesis of secondary metabolites” and “aminoacyl-tRNA biosynthesis” pathways were common. For the OC vs. NOC combination, the most highly represented pathways were “photosynthesis”, “plant hormone signal transduction”, and “terpenoid backbone biosynthesis” and for the PT–SH vs. PT combination they were “alanine, aspartate, and glutamate metabolism” and “biosynthesis of secondary metabolites”.

### Selection of OC, PT, and PT–SH-responsive genes

**Fig. 7:**
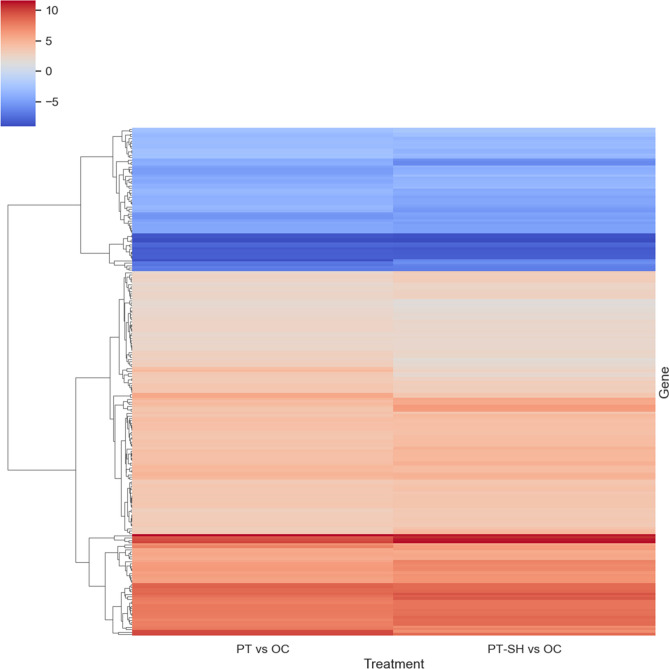
Heatmap plot using the hierarchical clustering method presenting the expression pattern of 216 common DEGs for PT vs. OC and PT–SH vs. OC; three main clusters are shown; expression values presented after being log-transformed; decreased and increased expression of DEGs are distinguished in blue and red, respectively

Among the 3028 DEGs identified for the OC vs. NOC combination, 252 DEGs were defined as uniquely expressed in OC (Fig. 5b, Suppl. data S2). The Venn diagram showing the overlap of DEGs among PT and PT–SH related to OC (Fig. 5c) identified 98 unique DEGs for PT vs. OC (Suppl. data S2) and 1305 unique DEGs for PT–SH vs. OC (Suppl. data S2). Among the 216 DEGs that were common in both combinations, i.e., PT vs. OC and PT–SH vs. OC, indicated genes that differed in the expression pattern (Suppl. data S2). The set of these common DEGs was subjected to hierarchical clustering (Fig. 7). The results showed that most of the DEGs presented a similar expression pattern between PT and PT–SH related to OC; however, DEGs with different expression levels are noticeable (Table 1). Among the DEGs whose expression was higher for the PT vs. OC combination than for PT–SH vs. OC were genes related to lipid transport (putative lipid-transfer protein DIR1), multicellular organism development (cytochrome P450 87A3), and regulation of growth (zeatin *O*-glucosyltransferase, calcium-binding protein PBP1), cell cycle (division) (protein ALP1-like, probable nicotinate-nucleotide adenylyltransferase), cell wall biogenesis (xyloglucan endotransglucosylase/hydrolase 2), and regulation of transcription (heat stress transcription factor A-3) (Table 1). The DEGs identified for the PT–SH vs. OC combination which characterized higher expression levels than for DEGs identified in the PT vs. OC combination were involved in multicellular organism development (protein light-dependent short hypocotyls, transcription factor MYB74, remorin 4,1), encoding actinidain (cysteine protease responsible for the cleavage of kiwellin), and the regulation of growth (3-ketoacyl-CoA synthase 6), lipid metabolism (linoleate 13S-lipoxygenase 2-1), and the carbohydrate metabolic process (uncharacterized protein RP505, beta-galactosidase 12) (Table 1). Finally, each type of examined callus—i.e., OC, PT, and PT–SH—was described by unique enriched GO terms for which were indicated unique genes expressed only in one type of callus (Table 2).

**Table 1:** Set of common DEGs identified for PT vs OC and PT–SH vs OC combinations with different expression level

Gene ID	Annotation	GO terms	Fold change*	
			PT vs OC	PT–SH vs OC
TRINITY_DN52519_c0_g1	Putative lipid-transfer protein DIR1	GO:0006869: biological process, lipid transport	9.77	6.96
TRINITY_DN76721_c1_g1	Cytochrome P450 87A3	GO:0016705:molecular function, oxidoreductase activity, acting on paired donors, with incorporation or reduction of molecular oxygen GO:0007275:biological process, multicellular organism development	7.63	6.31
TRINITY_DN75065_c0_g4	14 kDa proline-rich protein DC2	GO:0016021: cellular component, integral component of membrane	5.74	3.66
TRINITY_DN69939_c0_g2	Protein ALP1-like	GO:0071931:biological process, positive regulation of transcription involved in G1/S transition of mitotic cell cycle	5.63	3.80
TRINITY_DN79808_c0_g3	Calcium-binding protein PBP1	GO:0009733: biological process, response to auxin	4.72	2.76
TRINITY_DN77541_c2_g2	Protein NDR1	GO:0015986: biological process, ATP synthesis coupled proton transport	4.27	2.39
TRINITY_DN68307_c1_g1	Probable nicotinate-nucleotide adenylyltransferase	GO:0008017:molecular function, microtubule binding GO:0090235:biological process, regulation of metaphase plate congression	3.67	2.31
TRINITY_DN87714_c0_g1	Heat stress transcription factor A-3	GO:0006351:biological process, transcription, DNA-templated	3.49	1.96
TRINITY_DN84995_c6_g2	Xyloglucan endotransglucosylase/hydrolase 2	GO:0004553:molecular function, hydrolase activity, hydrolyzing *O*-glycosyl GO:0042546:biological process, cell wall biogenesis	3.04	1.81
TRINITY_DN93196_c5_g2	Zeatin *O*-glucosyltransferase	GO:0009965:biological process, leaf morphogenesis GO:0045926:biological process, negative regulation of growth GO:0010083:biological process, regulation of vegetative meristem growth GO:0010015:biological process, root morphogenesis	− 8.75	− 6.13
TRINITY_DN82049_c2_g5	Protein light-dependent short hypocotyls	GO:0007275:biological process, multicellular organism development GO:0090698:biological process, post-embryonic plant morphogenesis	10.04	11.40
TRINITY_DN84626_c2_g1	Uncharacterized protein RP505	GO:0016853: molecular function, isomerase activity GO:0005975: biological process, carbohydrate metabolic process	9.43	10.66
TRINITY_DN93276_c5_g3	**Linoleate 13S-lipoxygenase 2-1**	GO:0031408:biological process, oxylipin biosynthetic process	6.55	4.93
TRINITY_DN93876_c1_g1	S-ribosylhomocysteine lyase	GO:0005506:molecular_function, iron ion binding GO:0043768:molecular_function, S-ribosylhomocysteine lyase activity	5.80	7.55
TRINITY_DN80170_c3_g1	Homeobox protein SBH1	GO:0006355:biological process, regulation of transcription, DNA-templated	4.34	6.45
TRINITY_DN68675_c1_g2	3-ketoacyl-CoA synthase 6	GO:0006633:biological process, fatty acid biosynthetic process GO:0009826:biological process, unidimensional cell growth	4.17	5.19
TRINITY_DN58457_c0_g5	**Actinidain**	GO:0008234:molecular function, cysteine-type peptidase activity	3.88	6.31
TRINITY_DN86243_c0_g1	2-oxoglutarate-dependent dioxygenase DAO	GO:0009852:biological process, auxin catabolic process	3.85	6.19
TRINITY_DN63160_c0_g2	Transcription factor MYB74	GO:0030154:biological process, cell differentiation	3.80	5.16
TRINITY_DN73662_c6_g1	Remorin 4,1	GO:0009738:biological process, abscisic acid-activated signaling pathway GO:1900458:biological process, negative regulation of brassinosteroid mediated signaling pathway	3.16	4.42
TRINITY_DN93417_c5_g2	Beta-galactosidase 12	GO:0005975: biological process, carbohydrate metabolic process	− 3.83	− 5.94

Genes selected for qRT-PCR analysis are bolded

*In table set DEGs for which difference in Fold change between PT vs OC and PT–SH vs OC combinations was > 1

**Table 2:** Unique expressed genes in analysed kind of organogenic callus i.e. OC, PT and PT–SH. Genes selected for qRT-PCR analysis are bolded

GO term		Gene ID	Annotation	Fold Change		
				OC vs NOC	PT vs OC	PT–SH vs OC
*Uniquely enriched GO for OC with assigned transcripts:*						
Photosynthetic electron transport in photosystem I (GO:0009773)	TRINITY_DN57467_c1_g1		Photosynthetic NDH subunit of subcomplex B 3	6.2	–	–
	TRINITY_DN58289_c0_g1		Photosynthetic NDH subunit of subcomplex B 4	8.26	–	–
	TRINITY_DN88723_c0_g1		PGR5-like protein 1A	2.99	–	–
	TRINITY_DN72445_c3_g1		PGR5-like protein 1B	3.31	–	–
	TRINITY_DN64834_c0_g1		Photosystem I reaction center subunit V	4.28	–	–
	TRINITY_DN66548_c4_g1		NAD(P)H-quinone oxidoreductase subunit L	2.41	–	–
	TRINITY_DN68164_c0_g1		Protein proton gradient regulation 5	4.28	–	–
	TRINITY_DN73301_c0_g1		Protein curvature thylakoid 1B	7.26	–	–
Photosystem II assembly and stabilization (GO:0042549, GO:0010207)	TRINITY_DN77789_c2_g1		Photosystem II repair protein PSB27-H1	10.26	–	–
	TRINITY_DN58362_c2_g1		Photosystem II 10 kDa polypeptide	9.32	–	–
	TRINITY_DN73073_c3_g4		Photosystem II reaction center protein	4.85	–	–
	TRINITY_DN73899_c0_g1		Oxygen-evolving enhancer protein 1	10.16	–	–
	TRINITY_DN70457_c1_g1		Protein MET1	4.98	–	–
	TRINITY_DN78745_c2_g1		Chlorophyll a-b binding protein CP26	5.22	–	–
1-Deoxy-d-xylulose 5-phosphate biosynthetic process (GO:0052865)	TRINITY_DN76584_c0_ g2		Chaperone protein dnaJ C76	9.10	–	–
	TRINITY_DN72056_c0_g1		Probable 1-deoxy-d-xylulose-5-phosphate synthase	2.10	–	–
Negative regulation of brassinosteroid biosynthetic process (GO:0010423)	TRINITY_DN86056_c0_g1		BRI1 kinase inhibitor	6.68	–	–
Positive regulation of development (GO:0045962)	TRINITY_DN69303_c1_g1		Transcription factor TCP4	5.66	–	–
	TRINITY_DN72246_c1_g4		Transcription factor TCP2	4.99	–	–
	TRINITY_DN89003_c0_g1		Transcription factor TCP3	2.08	–	–
Somatic embryogenesis (GO:0010262)	TRINITY_DN56958_c0_g1		Endochitinase EP3	2.83	–	–
	TRINITY_DN75348_c1_g1		Nuclear transcription factor Y subunit A-5	9.47	–	–
	TRINITY_DN92178_c0_g1		Nuclear transcription factor Y subunit A-6	5.80	–	–
	TRINITY_DN80314_c1_g1		Nuclear transcription factor Y subunit A-1	3.29	–	–
Stem vascular tissue pattern formation (GO:0010222)	TRINITY_DN59946_c2_g4		ABC transporter G family member 14	11.09	–	–
Lignan biosynthetic process (GO:0009807)	TRINITY_DN72779_c1_g5		Secoisolariciresinol dehydrogenase	6.88	–	–
	TRINITY_DN74720_c1_g3		Momilactone A synthase	6.33	–	–
Acylglycerol lipase activity (GO:0047372)	TRINITY_DN56637_c0_g2		Patatin-like protein 3	3.82	–	–
	TRINITY_DN69630_c5_g1		Patatin-like protein 2	8.53	–	–
Carbohydrate transmembrane transporter activity (GO:0015144)	TRINITY_DN58919_c1_g4		Sugar transport protein MST4	2.96	–	–
	TRINITY_DN65561_c0_g1		Sucrose transport protein	4.36	–	–
	TRINITY_DN93668_c2_g1		Bidirectional sugar transporter SWEET12	6.91	–	–
	TRINITY_DN94065_c1_g1		Sugar transporter ERD6-like 16	3.76	–	–
	TRINITY_DN69374_c0_g1		Sucrose transport protein SUC1	2.54	–	–
	TRINITY_DN73457_c0_g1		Sugar transport protein 14	1.79	–	–
	TRINITY_DN74054_c4_g1		Probable inositol transporter 2	6.04	–	–
	TRINITY_DN76447_c2_g1		Triose phosphate/phosphate translocator TPT	2.42	–	–
	TRINITY_DN76650_c0_g2		Protein MKS1	3.81	–	–
	TRINITY_DN78448_c4_g1		Sugar carrier protein A	2.74	–	–
	TRINITY_DN80072_c1_g3		D-xylose-proton symporter-like 3	2.42	–	–
	TRINITY_DN86702_c0_g1		Probable polyol transporter 6	3.04	–	–
	TRINITY_DN87464_c0_g1		Ascorbate transporter	6.11	–	–
	TRINITY_DN89386_c0_g2		Probable anion transporter 4	3.98	–	–
Regulation of cutin biosynthetic process (GO:1901957)	TRINITY_DN63758_c3_g1		Ethylene-responsive transcription factor WRI1	− 7.38	–	–
	TRINITY_DN70719_c4_g1		AP2-like ethylene-responsive transcription factor	− 4.33	–	–
	TRINITY_DN84636_c3_g1		Transcription factor MYB16	− 2.02	–	–
*Uniquely enriched GO for PT with assigned transcripts:*						
Carbohydrate metabolic process (GO:0005975)	TRINITY_DN81835_c1_g1		**Beta-glucosidase 45**	–	8.98	–
Carbohydrate biosynthetic process (GO:0016051)	TRINITY_DN58086_c1_g1		Ethylene-responsive transcription factor ERF014	–	4.73	–
	TRINITY_DN74830_c2_g4		Ribulose bisphosphate carboxylase large chain	–	8.23	–
Starch biosynthetic process (GO:0019252)	TRINITY_DN55769_c0_g1		**Granule-bound starch synthase 1**	–	2.41	–
	TRINITY_DN90636_c2_g1		1,4-alpha-glucan-branching enzyme		2.31	
	TRINITY_DN83578_c0_ g1		Glucose-1-phosphate adenylyltransferase large subunit 1	–	3.44	–
	TRINITY_DN75074_c3_g1		Glucose-1-phosphate adenylyltransferase small subunit 2	–	2.56	–
Beta-glucan biosynthetic process (GO:0051274, GO:0051273) Multidimensional cell growth (GO:0009825)	TRINITY_DN50753_c0_g1		Cellulose synthase A catalytic subunit 9 [UDP-forming]	–	2.51	–
	TRINITY_DN67713_c2_g1		Cellulose synthase A catalytic subunit 6 [UDP-forming]	–	2.41	–
	TRINITY_DN86114_c0_g1		Cellulose synthase A catalytic subunit 8 [UDP-forming]	–	2.35	–
	TRINITY_DN59345_c1_g1		Cellulose synthase A catalytic subunit 3 [UDP-forming]	–	2.00	–
	TRINITY_DN75074_c3_		Glucose-1-phosphate adenylyltransferase small subunit 2	–	2.56	–
	TRINITY_DN94115_c1_g2		Cellulose synthase A catalytic subunit 2 [UDP-forming]	–	2.56	–
	TRINITY_DN83578_c0_g1		Glucose-1-phosphate adenylyltransferase large subunit 1	–	3.44	–
	TRINITY_DN90636_c2_g1		1,4-alpha-glucan-branching enzyme	–	2.31	–
	TRINITY_DN91058_c1_g5		Protein COBRA	–	2.51	–
Suberin biosynthesis process (GO:0010345)	TRINITY_DN76911_c0_g3		**Glycerol-3-phosphate acyltransferase 5**	–	7.61	–
Xyloglucan 1,6-alpha-xylosidase activity (GO:0080176)	TRINITY_DN53504_c0_g1		Alpha-xylosidase 1		− 4.70	
Glycerate dehydrogenase activity (GO:0008465)	TRINITY_DN92733_c1_g1		Glycerate dehydrogenase		− 3.93	
*Uniquely enriched GO for PT–SH with assigned transcripts:*						
Maintenance of shoot apical meristem identity (GO:0010492)	TRINITY_DN69495_c0_g4		**Regulatory-associated protein of TOR 1**	–	–	9.15
Epidermal cell differentiation (GO:0009913)	TRINITY_DN62776_c3_g1		Homeobox-leucine zipper protein, protodermal factor 2	–	–	7.46
Response to abscisic acid (GO:0009737), response to lipid (GO:0033993)	TRINITY_DN47952_c1_g1		Protein LHY	–	–	1.75
	TRINITY_DN56137_c0_g1		G-box-binding factor 3	–	–	2.23
	TRINITY_DN72762_c0_g2		ABSCISIC ACID-INSENSITIVE 5-like protein 5	–	–	1.78
	TRINITY_DN63160_c0_g2		Transcription factor MYB74	–	–	1.37
	TRINITY_DN63404_c2_g3		Non-specific lipid-transfer protein 3	–	–	5.13
	TRINITY_DN64420_c2_g1		bZIP transcription factor TRAB1	–	–	1.95
	TRINITY_DN71104_c0_g1		Homeobox-leucine zipper protein ATHB-7	–	–	3.80
	TRINITY_DN72973_c1_g1		Ethylene-insensitive protein 2	–	–	1.28
	TRINITY_DN74098_c7_g1		Ninja-family protein AFP2	–	–	3.75
	TRINITY_DN89649_c0_g4		Type IV inositol polyphosphate 5-phosphatase 11	–	–	2.67
	TRINITY_DN76822_c0_g1		Protein phosphatase 2C	–	–	1.85
	TRINITY_DN78984_c0_g2		Protein ASPARTIC PROTEASE IN GUARD CELL 1	–	–	7.73
	TRINITY_DN81281_c1_g4		E3 ubiquitin-protein ligase AIRP2	–	–	2.40
	TRINITY_DN44414_c0_g1		WD repeat-containing protein 78	–	–	8.37
	TRINITY_DN67609_c2_g1		Serine carboxypeptidase-like 25	–	–	8.0
	TRINITY_DN86821_c0_g1		Zinc finger protein 4	–	–	1.29
Transport of water (GO:0006833), Water channel activity (GO:0015250)	TRINITY_DN64148_c5_g3		Probable aquaporin PIP2-5	–		2.19
	TRINITY_DN67435_c5_g1		Probable aquaporin TIP1-1	–		1.65
	TRINITY_DN83985_c0_g3		LRR receptor-like serine/threonine-protein kinase GSO1	–		1.43
	TRINITY_DN71575_c0_g5		Aquaporin TIP2-1	–	–	− 2.0
Transcription regulator activity (GO:0140110)	TRINITY_DN76290_c2_g1		B-box zinc finger protein 32	–	–	3.13
	TRINITY_DN78978_c0_g1		Cyclic dof factor 3 E	–	–	2.82
	TRINITY_DN79556_c1_g2		NAC domain-containing protein 21/22	–	–	2.65
	TRINITY_DN80189_c2_g1		Dof zinc finger protein DOF5,4	–	–	1.54
	TRINITY_DN81175_c0_g2		ABSCISIC ACID-INSENSITIVE 5-like protein 5	–	–	1.35
	TRINITY_DN84340_c2_g1		Nuclear transcription factor Y subunit A-1	–	–	4.21
	TRINITY_DN84757_c2_g1		Transcriptional activator TAF-1	–	–	1.71
	TRINITY_DN87520_c1_g2		B-box domain protein 31	–	–	1.98
	TRINITY_DN88759_c2_g2		Transcriptional repressor ILP1	–	–	7.88
	TRINITY_DN89482_c1_g4		Common plant regulatory factor 1	–	–	2.06
	TRINITY_DN93263_c3_g1		Floral homeotic protein APETALA 2	–	–	1.81
	TRINITY_DN84542_c0_g2		BTB/POZ and TAZ domain-containing protein 2	–	–	− 1.86
Symporter activity (GO:0015293)	TRINITY_DN62856_c0_g1		Amino acid permease 4	–	–	2.46
	TRINITY_DN89229_c1_g1		Esculentin-2CG1	–	–	1.37
	TRINITY_DN89293_c1_g5		Amino acid permease 3			2.38
Hemicellulose metabolic process (GO:0010410)	TRINITY_DN67175_c0_g1		Probable xyloglucan endotransglucosylase/hydrolase protein 7	–	–	3.80
	TRINITY_DN77522_c0_g1		Xyloglucan endotransglucosylase/hydrolase protein 9	–	–	2.55
	TRINITY_DN79364_c1_g3		Probable xyloglucan endotransglucosylase/hydrolase protein 32	–	–	1.99
	TRINITY_DN80529_c4_g2		Probable xyloglucan endotransglucosylase/hydrolase protein 23	–	–	− 2.34
	TRINITY_DN92346_c1_g3		Probable xyloglucan endotransglucosylase/hydrolase protein 26	–	–	− 2.99
	TRINITY_DN78783_c3_g2		Probable xyloglucan endotransglucosylase/hydrolase protein 28	–	–	− 1.52
	TRINITY_DN82331_c3_g1		Beta-xylosidase/alpha-L-arabinofuranosidase 2	–	–	− 1.74

Among the pool of expressed genes in the OC were genes involved in photosynthesis and chloroplast metabolism. Eight up-regulated genes were involved in photosynthetic electron transport in PSI: photosynthetic NDH subunit of subcomplex B3 and B4 (LogFC = 6.2–8.3), encoding protein curvature thylakoid 1B (LogFC = 7.3), genes of PSI reaction center subunit V, and encoding protein proton gradient regulation 5 (LogFC = 4.3); six genes connected with the stabilization and assembly of PSII were significantly activated: PS II repair protein PSB27-H1 (LogFC = 10.3) and oxygen-evolving enhancer protein 1 (LogFC = 10.3), while gene encoded chaperone protein dnaJ C76 was strongly activated (LogFC = 9.1). In OC, we found that the expression of genes regulating the developmental process and transcription factors mainly dominated, i.e., TCP2-4 (LogFC = 2.1–5.7), nuclear transcription factor Y subunit A-1, A-5, and A-6 (LogFC = 3.3–9.5), but high expression (LogFC = 11.1) was also observed for ABC transporter G family member 14 gene, which is involved in stem vascular tissue pattern formation. Moreover, BR11 kinase inhibitor gene—related to the brassinosteroid biosynthetic process—was found to be up-regulated in relation to NOCs (LogFC = 6.7). In OCs, we identified DEGs that are associated with the lignan biosynthetic process: encoded secoisolariciresinol dehydrogenase (LogFC = 6.9) and momilactone A synthase (LogFC = 6.3). There were numerous DEGs associated with carbohydrate transmembrane transport, such as bidirectional sugar transporter SWEET12 (LogFC = 6.9), probable inositol transporter 2 (LogFC = 6.1), and ascorbate transporter (LogFC = 6.1). In OC we also identified down-regulated TF genes that were associated with the regulation of cutin biosynthetic process: ethylene-responsive TF WRI1 (LogFC = -7.4) or AP2-like ethylene-responsive TF (LogFC = − 4.3) (Table 2). In PT, unique DEGs were mostly related to carbohydrate metabolism and were associated with growth and developmental processes and cell wall structure. The beta-glucosidase 45 gene was found to be highly up-regulated (LogFC = 9.0), as were genes associated with starch biosynthetic process: granule-bound starch synthase 1 gene, glucose-1-phosphate adenylyltransferase genes, and genes which encode cellulose synthase subunits (Table 2). Among unique DEGs involved in cell wall modification, glycerol-3-phosphate acyltransferase 5 gene was activated in PT (LogFC = 7.6), while alpha-xylosidase 1 gene was down-regulated (LogFC = -4.7). In PT–SH the expression of genes involved in developmental processes and response to plant growth regulators was observed: ABA and lipids, transport of substances, cell wall modification, and secondary metabolites biosynthesis; numerous transcription factors were also activated (Table 2). Among unique DEGs for PT–SH, especially high expression levels were reported for genes such as regulatory-associated protein of TOR 1, which is associated with the maintenance of shoot apical meristem identity (LogFC = 9.1), and homeobox-leucine zipper protein, protodermal factor 2, which is involved in epidermal cell differentiation (LogFC = 7.5). Interestingly, among six genes annotated as xyloglucan endotransglucosylase/hydrolase protein, three of them were up-regulated (TRINITY_DN67175_c0_g, TRINITY_DN77522_c0_g1, and TRINITY_DN79364_c1_g3) while three were down-regulated (TRINITY_DN80529_c4_g2, TRINITY_DN92346_c1_g3, and TRINITY_DN78783_c3_g2) (Table 2).

### RT-qPCR expression analysis of OC, PT, and PT–SH-responsive genes involved in carbohydrate, lipid and second metabolites metabolism

**Fig. 8:**
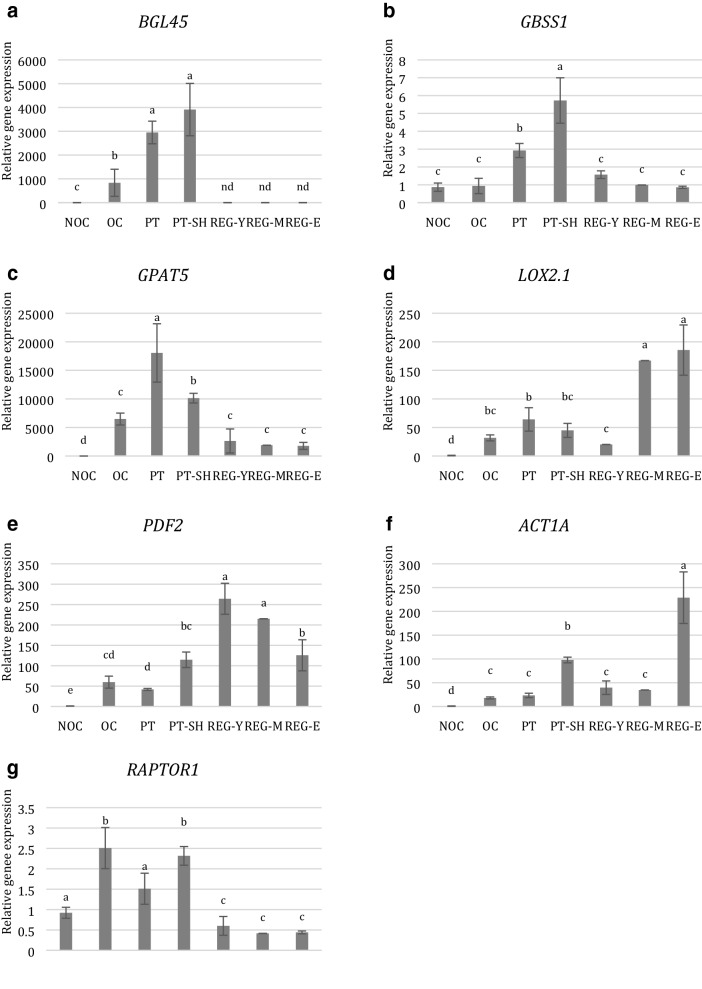
Relative expression levels of *BGL45* (**a**), *GBSS1* (**b**), *GPAT5* (**c**), *LOX2.1* (**d**), *PDF2* (**e**), *ACT1A* (**f**), and *RAPTOR1* (**g**) genes for cultured explants—NOC, OC, PT, PT–SH, and regenerated plants at different ages: REG-Y, REG-M, REG-E in *Actinidia chinensis* cv. *deliciosa*. Error bars show the mean standard error calculated from three independent biological replicates. The same letters indicate a lack of differences in relative expression levels between developmental stages; *p* < 0.05; *nd* not determined in qRT-PCR

The results showed that, compared with NOC, all of the examined genes (Table S1) appeared to be up-regulated in different regions of OC–PT and PT–SH (Fig. 8). Genes related to carbohydrate metabolism, i.e., beta-glucosidase 45 (*BGL45*) and granule-bound starch synthase 1 (*GBSS1*), had differential expression patterns among the investigated developmental stages. An increase in the expression of the *BGL45* gene was noted in OC; however, an expression three times higher was evidenced in PT–SH, though no significant differences in transcript abundance were detected between PT and PT–SH (Fig. 8a). Interestingly, the expression of this gene was not determined in regenerated plants. The greatest increase in *GBSS1* gene activity was evidenced for PT with shoots (PT–SH), it was two times higher than in PT, although no significant differences in the expression of that gene were noted between NOC and OC or all REGs (Fig. 8b). For genes involved in the suberin biosynthesis process—glycerol-3-phosphate acyltransferase (*GPAT5*)—and lipid metabolism—linoleate 13 s-lipoxygenase 2-1 (*LOX2.1*)—differences in the expression levels of *GPAT5* were observed in all probes, wherein the expression profile was as follows: PT > PT–SH > OC = REG > NOC (Fig. 8c). The greatest increase in *LOX2.1* gene activity was observed in regenerated plants at the medium and elderly stages (REG-M, REG-E)—it was about 2.5 times higher than in OC and PT–SH (Fig. 8d). Differential expression patterns for homeobox-leucine zipper protein protodermal factor 2 (*PDF2*) gene, which are related to epidermal cell differentiation, were found across PT–SH and PT, wherein about a threefold increase in PT–SH was observed (Fig. 8e). The age of regenerated plants had a considerable influence on the expression of the *PDF2* gene, i.e., a statistically significantly lower gene expression was observed in REG-E in comparison to REG-Y and REG-M. The increase in the expression of the actinidain (*ACT1A*) gene was evidenced in PT–SH (Fig. 8f) in relation to OC and PT, but the highest expression level was indicated in elderly regenerated plants (REG-E). The expression profile of the gene related to the organization of the apical meristem of shoots, i.e., regulatory associated protein of TOR1 (*RAPTOR1*), was interesting (Fig. 8g): an increase in transcript abundance was noted in OC and PT–SH, whereas no difference in expression was detected between PT and NOC, and the inhibition of *RAPTOR1* expression was observed in all regenerants probes. To sum up, each region of OC could be characterized by the highest expression levels of specific genes evaluated in comparison to NOC—i.e., *GPAT5* for PT and *GBSS1*, *PDF2*, and *ACT1A* for PT–SH—and in general, the expression profiles of *BGL45*, *LOX2.1*, and *RAPTOR1* genes were common for OC, PT, and PT–SH.

## Discussion

### Morphological structures and histological organization of PT

The long-term callus cultures can be regarded as a more homogenous tissue following the loss of regeneration capacity than short-term cultures (Bajaj and Rajam 1996; Gondo et al. 2017; Feher 2019). Despite this disadvantage, data on the features of calluses more than one year old and their efficiency of plant regeneration by somatic embryos or adventitious shoot bud development have been reported, for example, for *Oryza* (Bajaj and Rajam 1996), *Asparagus* (Pontaroli and Camadro 2005), *Coffea* (Landey et al. 2015), *Fagopyrum* (Betekhtin et al. 2017), and *Pennisetum* (Gondo et al. 2017). In the case of kiwifruit, a two-year-old white, friable callus was used for the protoplast culture and then shoot regeneration via organogenesis (Oliveira and Pais 1991).

Histological and ultrastructural analysis of the four- and eight week-old endosperm-derived callus in *Actinidia* revealed that the regeneration processes appeared as bulges near the remnants of endosperm tissue (Popielarska-Konieczna et al. 2011). In a long-term culture, PTs were forming continuously within the organogenic, green callus. It is worth noting that only one kind of plant growth regulator, thidiazuron, was used for the whole period of culturing—from the inoculation of the endosperm explant and callus induction through the four years of callus culturing. The most distinct differences between the bulges from the initial and long-term cultures are the size and shape of the structures, and the organization of their surface (a single cell layer typical of the epidermis-like layer versus a layer several cells thick in the outer part of a PT).

Structures, described as protrusions or outgrowths of the callus, have been observed during tissue culturing of different explants and species. The morphology, especially the shape of the PTs in kiwifruit examined here, seems to be similar to the protocorm-like bodies in *Dendrobium* (Zhao et al. 2008) and *Phalaenopsis* (Chen et al. 2019).

The histological sections revealed differences in cell organization within OC from the long-term culture of kiwifruit. The regular cell composition on the surface in comparison with regions beyond the PT, the presence of starch granules, and the shoot buds arising on the PT can be mentioned as the most relevant. These features are similar to the histology of protocorm-like bodies or the multiple meristem-like tissues observed in Orchidaceae (Zhao et al. 2008; Chen et al. 2019) and nodular cultures in Bromeliaceae (Corredor-Prado et al. 2015). However, the histological organization of PT in kiwifruit also resembles PT observed in other woody vines—*Passiflora edulis* (Fernando et al. 2007). The crucial difference between the above-mentioned structures and PTs in kiwifruit is the modified secondary cell wall of the majority of several-cell layers, whereas for the protocorm-like bodies and nodular structures a single-cell layer is typically reported. Because of this feature, PT observed in long-term callus in kiwifruit show some similarity to tumors, called crown-galls, which have been analyzed in *Arabidopsis thaliana* (Efetova et al. 2007).

### Relationship between the morpho-ultrastructural features, transcriptomic profiles, and the expression of OC, PT, and PT–SH–responsive genes

Transcriptome profiling is a useful tool for distinguishing structures with morphological similarity and to confirm their different nature (Chen et al. 2019). Additionally, the gene expression during callus induction and proliferation, as well as the regeneration processes from callus, and then de novo shoot bud regeneration, has received more and more attention from researchers (Nakano et al. 2018; Fu et al. 2019; Gao et al. 2019; Ikeuchi et al. 2019 and references therein; Lee and Huang 2019, Ouyang et al. 2020).

In this study we demonstrated that many genes were up- or down-regulated within the callus and its region, so we filtered them out and selected the genes that were related to PT, PT–SH structures, and regions of OC without visible PT. We found that for OC the most active genes are involved in photosynthesis. The activity of genes involved in a negative regulation of the brassinosteroid biosynthetic process has an impact on photomorphogenesis (Halliday and Fankhauser 2003). Interestingly, some genes involved in the development are correlated with somatic embryogenesis. In calluses, especially in long-term callus cultures, a vascular tissue is often formed, which could be connected with the up-regulation of genes involved in stem vascular tissue pattern formation and the lignan biosynthetic process.

We found that the PT gene expression is predominantly in carbohydrate metabolism, cell growth and cell wall organization, and secondary metabolite biosynthesis. Several genes involved in the carbohydrate metabolism pathways in the PTs were strongly up-regulated, for example, the genes involved in starch synthesis. The initiation of organogenesis required an accumulation of storage substances, and energy mobilization has been reported to play an important role in shoot formation (Carciofi et al. 2012; Santos et al. 2018; Lee and Huang 2019). The expression of genes connected with modifying the existing cell wall and its integrity (Shigeyama et al. 2016) together with suberin biosynthesis, suggest the reorganization of cells towards the formation of other types of structures.

In PT–SH, the up-regulation of genes involved in the maintenance of shoot apical meristem identity and epidermal cell differentiation could be connected with shoot bud formation. Additionally, the high expression of genes involved in transporting substances (water, nitrogen, urea, and phosphate) is crucial during the growth of organs. Interestingly, the up-regulation of genes involved in regulating transcriptomic processes was observed, which suggests the high activity of cells and a modification of their metabolism (Ruttink et al. 2007; Lantzouni et al. 2020).

Among common up-regulated genes unique for PT and PT–SH, the actinidain gene, which is involved in cysteine-type peptidase activity, was noted. The high expression of this gene, involved in the synthesis of a protein specific to the genus Actinidia, could indicate that these structures have a transitional nature between callus and organ, like a leaf or shoot bud.

The relative expression of unique genes was analyzed with the qRT-PCR method. To understand the biological processes in which the selected seven genes are involved, a GO search was performed, revealing important roles related especially to carbohydrate metabolism and secondary metabolite biosynthesis and development.

### Distribution of starch granules in PT coincides with high *GBSS1* gene expression

Starch is the most universal form of carbon reservoir in plants, for energy and substrates. This insoluble polymer is synthesized inside plastids in higher plants (Martin and Smith 1995; Geigenberger 2011). In the present study, plastids with starch granules were observed in the cells of PTs, peripherally outgrowing structures within the OC. The high accumulation of starch in cultured tissue visible as starch granules was mainly noted in regions where shoot buds ultimately formed, for example, in stem explants in *Begonia* (Mangat et al. 1990) or organogenic callus in *Vanilla* (Palama et al. 2010). The deposition of starch granules in callus of different species might serve as an energy reserve for the energy-consuming organogenic processes such as somatic embryogenesis or shoot meristem development (Carciofi et al. 2012). Moreover, it has been found that in the culture system of callus in rice, the sucrose uptake and distribution of starch granules coincides with the regeneration of plants (Lee and Huang 2019). Interestingly, the accumulation of starch granules was observed in the peripheral regions of rice callus.

Changes in carbohydrate metabolism accompanied by higher mRNA transcription levels of the genes involved in starch synthesis have been found, e.g., in the embryogenic callus in *Picea balfouriana* (Li et al. 2014) and the organogenic, highly regenerable callus in rice (Lee and Huang 2019). Increased expression of *GBSS1* was detected in the embryogenic callus in oil palm (Santos et al. 2018). GBSS1 enzyme is required for the starch biosynthesis pathway, especially for the elongation of the amylose polymer, and it is closely associated with the formation of starch granules. The corresponding gene is highly conserved and is expressed in the whole plant, though mainly at the higher level, in leaves (Tenorio et al. 2003; Seung et al. 2015). We found that the expression of the *GBSS1* gene is significantly higher in PTs than in the other area of OCs, and even in the leaves of regenerated plants. This data corresponds with the histological and TEM observations, which did not reveal any plastids with starch granules in the callus cells beyond the PT.

We noted the presence of some electron-dense inclusions in plastids, which have been reported for different plant tissues. The study of etio-chloroplast development in *Nicotiana* revealed that these dark materials contain iron and phosphorus (Sprey et al. 1977). Some researchers have suggested the similarity of electron-dense materials inside plastids to ferritin protein clusters (Voith von Voithenberg et al. 2019). Electron-dense inclusions were also detected in the mesophyll of *Rosmarinus officinalis* during etio-chloroplast differentiation (Böszörményi et al. 2020). Altogether, the electron-dense inclusions in chloroplasts within PT and OC might indicate that the chloroplast development is still in progress. Moreover, we observed some plastids with an elongated shape, which have been found especially in plant tissue culture (Poljuha et al. 2003; Pavoković et al. 2012).

### Expression of *BGL45* and *GPAT5* suggest the presence of phenols and cell wall lipophilic polymers

Primary plant metabolites are those chemical compounds which are required for basic physiological processes. Thus, they are more or less similar in all plant cells. The products of subsidiary pathways are called secondary plant metabolites and can be species-specific; this is regarded as an adaptive capacity, which allows the plant to mitigate biotic and abiotic environmental stresses (Isah 2019). Phenolics, oxylipins, and lipophilic compounds are examples of the numerous secondary plant metabolites.

Phenolic inclusions can be visualized in plant tissues using different reagents, such as TBO and PAS staining (Gutmann 1995). Under TEM observations, the deposits might be identified as tannins, especially when they are visible as uniformly osmophilic materials adjoined to the inner side of the tonoplast in vacuoles (Brillouet et al. 2014). On the other hand, the extraction and analysis of bioactive compounds from the leaves of *Actinidia* indicate a significant amount of flavonoids (Henriques et al. 2018). In this study, the deposition of phenols was observed in both PTs and other regions of the long-term cultured organogenic callus using histochemical and TEM techniques. The phenol content might increase in tissues as a response to the culture conditions, as has been detected for cotton callus (Bibi et al. 2017), or to the culture age, as has been observed in a long-term culture of callus in *Fagopyrum tataricum* (Betekhtin et al. 2017).

A cuticle layer and suberin lamellae covering the outermost surface of plants are commonly known as the plant lipophilic barriers with a protective function against water loss and pathogen infection (Cohen et al. 2017). Their presence is associated with specialized cell types, such as the epidermis in primary organs, and the periderm during the secondary growth of the stem. Additionally, suberin was detected in the outer cell layers of tumors, called crown galls, whose structures are induced by *Agrobacterium* infection (Efetova et al. 2007). Auramine O shows a strong affinity to materials containing acids and unsaturated waxes, like cutin, lignin, suberin, and sporopollenin (Ursache et al. 2018). A previous study on organogenesis in kiwifruit was conducted on explants after a few weeks of culturing and the formation of adventitious shoot buds was noted (Popielarska et al. 2006). New shoots arise from the outer part of protuberances, which possess an epidermis-like tissue covered with a cutin layer on the outer surface of the cell wall (Popielarska-Konieczna et al. 2011). This layer was distinctly fluoresced after being stained with auramine O. However, in the present study, the surface cells in the PTs from the long-term culture show fluorescence in the cell walls, not a layer on the cells, which indicates the presence of suberin or lignin. It is worth noting that on the surface of the PTs was several layers of cells, not the single cell layer which is typical for epidermis. The presence of suberin can be visualized by other procedures, such as Sudan III staining. In this study, using this dye revealed the suberin deposits on the surface cells of PT. The modification of cell walls was reported for cultured carrot explants (Heale and Sharman 1977). Interestingly, lignin synthesis was detected in the surface cells of the callus, whereas slices of carrot roots showed the synthesis of suberin. The electron-opaque and electron-translucent layers of the suberin within the cell wall are very regular under TEM analysis, though the changes in the structure and thickness might be due to the species and growing conditions (Nawrath 2002). On the electronograms, the lamellae within the cell wall of the outer cell layers of PTs are visible, but they are not very regular.

In plants, beta-glucosidase (BGL) proteins are required for the hydrolysis of beta-glucosidic bonds between aglycone and glycone moieties, which is the mechanism for activating secondary metabolites. *BGL45* is annotated to the lignin and phenolic biosynthetic processes and is expressed in stems (Chapelle et al. 2012). A few reports concerning the transcriptomic analysis of calluses have reported down-regulation of genes which play a role in cell wall modification, such as lignification (Kumari et al. 2017). The data obtained for the callus in *Actinidia* confirm this finding. However, the higher expression of the *BGL45* in the PTs than in other regions of OC suggests that the tissue differentiation associated with organogenesis has been induced in PTs. Other enzymes, GPATs, are crucial in the glycerolipid pathways and are involved in a wide range of physiological processes, such as the synthesis of extracellular protective layers or the control of the chilling tolerance in plants (Li et al. 2018). One of them, GPAT5, is especially involved in the biosynthesis of suberin (Chen et al. 2011). Intriguingly, the highest expression of the *GPAT5* gene was found in PTs, especially those with no visible shoot buds. The modification of the protuberance surface with suberin might be an indicator of the age of these structures, due to the lower capacity to produce shoots, as has been reported for enlarged protuberances in *Passiflora* (Fernando et al. 2007).

### Expression of genes involved in signaling and development processes

The complex of TOR protein kinases are potent cell growth regulators in all eukaryotes. For plants, RAPTOR1 is especially considered to be the most crucial for promoting cell expansion and coordinating multicellular growth in response to external signals, such as nutrients and PGRs (Anderson and Hanson 2005; McCready et al. 2020). In the present study, higher expression (according to RNA-seq analysis) of the *RAPTOR1* gene was found in PT–SH. Lower expression of this gene, though still higher than in the leaves of regenerated plants, was noted in both the OC and PT. The high expression of *RAPTOR1*, called the “master regulator” (McCready et al. 2020) may indicate the high activity of developmental processes which take place in PT–SH. Contradictory results have been reported for the callus and regenerated shoots in tomato plants (Kumari et al. 2017).

Lipoxygenase (LOX) is a large family of plant enzymes that catalyze the hydroperoxidation of free polyunsaturated fatty acids into active substances called oxylipins (Wasternack and Feussner 2018 and references therein). The function of LOX is especially related to the plant’s defense against biotic and abiotic factors as well as plant development. The protein linoleate 13S-lipoxygenase 2-1 (LOX2.1) is located in chloroplasts within leaves and is considered to be the major form that provides the substrate for oxylipin (like jasmonate) biosynthesis (Wasternack and Feussner 2018). LOX2-1 from mesophyll cells was found to be an important factor in stomatal closure in *A*. *thaliana* (Sun et al. 2015). Under the plant tissue culture conditions, the LOX2 protein is involved in the induction of somatic embryogenesis, e.g., in *A*. *thaliana* (Mira et al. 2016). In the present study, we noticed a lower expression of *LOX2.1* in the OCs than in the PT and PT–SH. The results of qPCR show a higher expression of that gene in plants after a few months and after two years of regeneration in comparison with young regenerants. This finding may stem from incompletely developed chloroplasts appearing in the cultured tissue in this study.

The class IV homeodomain-leucine zipper protein family represents plant-specific transcription factors. One of them, the protein PDF2, is crucial for the differentiation and maintenance of shoot epidermal cells in *Arabidopsis* (Takada and Iida 2014). Together with its closest homologue, *Arabidopsis thaliana* meristem layer 1 (ATML1), induces the epidermal gene expression and epidermis-related traits, such as stomatal guard cell and trichome-like cell formation (Palovaara et al. 2016). In the present study, high *PDF2* expression was detected for PT–SH, which may be the result of the intensive growth of organs covered with the epidermis. Interestingly, the qRT-PCR analysis indicates that the gene expression level in both of 2-year-old plants and PT–SH was similar.

### *Actinidia*-specific protein

Actinidin (or actinidain [Act c1]) belongs to a large protein family, papain-like cysteine proteases (Bublin 2013), and is one of the 13 allergens identified in green (*A*. *chinensis* var. *deliciosa* formerly *A*. *deliciosa*) and golden (*A*. *chinensis*) kiwifruits (Nilsson et al. 2015). Actinidin is encoded by the *ACT1A* gene, which is expressed especially in the fruit of *A*. *deliciosa* cv. Hayward (Nieuwenhuizen et al. 2012). The extraction and electrophoresis analysis of proteins were conducted on seeds, fruits, and leaves in kiwifruit (Miraghaee et al. 2011). The results suggest that actinidin was expressed more in the fruit than in the leaves. These data are in agreement with previous studies conducted on *A*. *chinensis* (Praekelt et al. 1988), which indicated that the actinidin expression is largely restricted to the fruit. No data concerning the content of this protein or the expression level of the *ACT1A* gene in a callus have been found. In the present study, RNA-seq data confirmed the expression of the *ACT1A* gene in PT and PT–SH, though a higher expression level was observed in PT–SH, which was validated by qPCR analysis. The fact that the highest expression of *ACT1A* was found in the leaves from two-year-old regenerants suggests that actinidin synthesis take place in fully developed organs.

## Conclusion

The findings confirm the distinct nature of PTs, which were examined at the morphological, histological, and molecular levels. The specific shape of the structures, the regular composition of the cell layers, the presence of plastids with starch granules, and the cell wall modification—which has not been detected beyond PT—might be indications of their distinctness, even in the absence of shoot bud meristems. Although PT and especially PT–SH can be easily discriminated by macroscopic and microscopic observations, bioinformatics tools provide a powerful approach to distinguish the structures of PT within OC. We found gene expression patterns in PT and PT–SH related to development and metabolic pathways (starch and secondary metabolite biosynthesis). The high expression of the genes *BGL45*, *GPAT5*, and *GBSS1* coincides with the ultrastructural traits of PT cells and might be markers of these structures. Interestingly, *RAPTOR1*—the gene involved in multicellular development—seems to be crucial for PT–SH, when shoot bud formation was observed within PT. In line with these results, we suggest that PT are a kind of callus structure and a transitional stage before organogenesis. Therefore, the present study can contribute to the knowledge about the organization of callus, mass of pluripotent cells.

## Supplementary Information

Below is the link to the electronic supplementary material.

Supplementary file1 Supplementary Tables S1 - S6 (DOCX 32 KB)

Supplementary file2 Suppl. Fig. 1 Longitudinal sections of detached PT (a–c) and OC (d, e) in *Actinidia chinensis* cv. *deliciosa*; TBO (a, d) and auramine O (b, c, e) staining. a The part of ball-shaped PT with the compact composition of cells (black arrows) on the surface. b White arrows indicate cells on the surface where the cell walls show fluorescence (b, c); notice the autofluorescence of cell walls (stars). d, e OC with the surface composed of loosely attached cells (black open arrows) which show autofluorescence (white open arrows) (TIF 7556 KB)

Supplementary file3 Suppl. Fig. 2 Magnification of longitudinal sections of detached PT in *Actinidia chinensis* cv. *deliciosa*; Sudan III staining. Arrows indicate cells on the surface with a reddish color of the cell walls. Stars show the crystals of dye in the background. Dotted lines indicate lines of the merged pictures (TIF 1399 KB)

Supplementary file4 Suppl. Data S1 GO and KEGG annotations of unigenes * Actinidia chinensis* cv. deliciosa (XLSX 1612 KB)

Supplementary file5 Suppl. Data S2 List of common and specific DEGs for OC, PT and PT-SH *Actinidia chinensis*. *deliciosa* (XLSX 9369 KB)

Supplementary file6 Suppl. Data S3 Gene Ontology Biological Process (XLSX 1253 KB)

Supplementary file7 Suppl. Data S4 Gene Ontology Molecular Function (XLSX 2728 KB)

Supplementary file8 Suppl. Data S5 Gene Ontology Cellular Compartments (XLSX 549 KB)

## Data Availability

The datasets generated and/or analyzed during the current study are available from the corresponding author upon reasonable request.

## Abbreviations

*18S*: 18S rRNA
*ACT1A*: Actinidain
*BGL45*: Beta-glucosidase 45
*GBSS1*: Granule-bound starch synthase 1
*GPAT5*: Glycerol-3-phosphate acyltransferase 5
*LOX2.1*: Linoleate 13S-lipoxygenase 2-1
NOC: Non-organogenic callus
OC: Organogenic callus
*PDF2*: Homeobox leucine zipper protein, protodermal factor 2
PGR: Plant growth regulator
PT: Protuberance
PT–SH: Protuberance with visible shoot buds/shoots
*RAPTOR1*: Regulatory associated protein of TOR (target of rapamycin)
REG-Y: Regenerated plant at an early/young stage, four to six weeks after transfer onto a half-strength MS
REG-M: Regenerated plant at a medium stage, six to eight months after transfer onto a half-strength MS
REG-E: Regenerated plants at an elderly stage, two years after transfer onto a half-strength MS
