# Supplementary material for: Protuberances are organized distinct regions of long-term callus: histological and transcriptomic analyses in kiwifruit
Source: Plant Cell Rep. 2021 Feb 5;40(4):637–65. doi: 10.1007/s00299-021-02661-0 (PMC7954764; doi:10.1007/s00299-021-02661-0)
Supplement: Supplementary file 1 — Supplementary file1 Supplementary Tables S1 - S6 (DOCX 32 KB) [file 299_2021_2661_MOESM1_ESM.docx]

| Symbol | Gene | Accession number | Functional annotation **with Trinotate (Haas et al. 2013)** | | Gene model in Kiwifruit Genome Database* | Primer sequence (5’-­‐>3’) | Product size  (bp) |
| --- | --- | --- | --- | --- | --- | --- | --- |
|  |  |  | Swiss.Prot BLASTX hit | KEGG hit |  |  |  |
| *BGL45* | Beta-glucosidase 45 | MT451976 | BGL45_ARATH | ath:AT1G61810 | Achn257451 | F: GGGACCGAGTTAAGTACTGG | 153 |
|  |  |  |  |  |  | R: GAGCAGCAACGAAAGGATCG |  |
| *GBSS1* | Granule-bound starch synthase 1 | MT451977 | SSG1_ANTMA | - | Achn045781 | F: TATGCCTGTGCTTCCGCAAT | 77 |
|  |  |  |  |  |  | R: GCAGGGAAGAAGAGCAGTGT |  |
| *GPAT5* | Glycerol-3-phosphate acyltransferase 5 | MT451975 | GPAT5_ARATH | ath:AT3G11430 | Achn257911 | F: GAGGGTGGTGATTCGGTCTC | 121 |
|  |  |  |  |  |  | R: AGGATTTTTCCTTGCACATAGAC |  |
| *LOX2.1* | Linoleate 13S-lipoxygenase 2-1 | MT451978 | LOX21_SOLTU | sot:102596122 | Achn123641 | F: GCATCCAGTTGGTAAAGGAATG | 84 |
|  |  |  |  |  |  | R: CTTCGGTGATGGCTGATTCT |  |
| *PDF2* | Homeobox-leucine zipper protein, protodermal factor 2 | MT460908 | PDF2_ARATH | ath:AT4G04890 | Achn257441 | F: TGGAATCTTTCTTCAAGGAGTGTC | 131 |
|  |  |  |  |  |  | R: CTTCGCCTTCATTTGGGTGC |  |
| *ACT1A* | Actinidain | MT463287 | ACTN_ACTCH | - | Achn336531 | F: GGGATTTGCTTGTCCTCCTAATA | 91 |
|  |  |  |  |  |  | R: ATGCGTGACTTAGAGGAATTGA |  |
| *RAPTOR1* | Regulatory-associated protein of TOR 1 | MT451979 | RTOR1_ARATH | ath:AT3G08850 | Achn388731 | F: TCTTCTTCGCAAGATCACGCT | 98 |
|  |  |  |  |  |  | R: GCACATACGAGGAATTCAGGGGT |  |
| *ACT1* | Actin** | EF063572.1 | - | - | - | F: GCTTACAGAGGCACCACTCAACC | 105 |
|  |  |  |  |  |  | R: CCGGAATCCAGCACAATACCAG |  |
| *18S* | 18S ribosomal RNA** | AF419792.1 | - | - | - | F: CTGTGAAACTGCGAATGGCTC | 110 |
|  |  |  |  |  |  | R: TTCCAGAAGTCGGGGTTTGT |  |
| * The Kiwifruit Genome Database, http://bioinfo.bti.cornell.edu/kiwi (Huang et al. 2013), ** Primer sequences for *act1* (Li et al. 2013), and for *18S* (Ferradás et al. 2016) | | | | | | | |

**Table S1** Gene sequence data and primers used in the analyses of cultured plant material in *Actinidia chinensis* cv. *deliciosa*.

**Table S2** *Actinidia chinensis* var. *deliciosa* transcriptome sequencing data

| Sample | Biological replication | Read length (bp) | # clean reads | Size of clean reads (Gbp) | GC% | Average per base sequence quality |
| --- | --- | --- | --- | --- | --- | --- |
| NOC | NOC1 | 100 +100 | 28,544,617 | 2.85 | 47 | Q39 |
|  | NOC2 | 100 +100 | 33,182,983 | 3.32 | 46 | Q39 |
|  | NOC3 | 100 +100 | 27,878,447 | 2.79 | 47 | Q39 |
| Total |  |  | 89,606,047 | 8.96 |  |  |
| OC | OC1 | 100 +100 | 33,753,783 | 3.37 | 47 | Q39 |
|  | OC2 | 100 +100 | 33,184,687 | 3.32 | 46 | Q39 |
|  | OC3 | 100 +100 | 32,756,429 | 3.27 | 46 | Q39 |
| Total |  |  | 99,694,899 | 9.96 |  |  |
| PT | PT1 | 100 +100 | 32,144,536 | 3.21 | 47 | Q39 |
|  | PT2 | 100 +100 | 27,810,020 | 2.78 | 47 | Q39 |
|  | PT3 | 100 +100 | 24,698,053 | 2.47 | 47 | Q39 |
| Total |  |  | 84,652,609 | 8.46 |  |  |
| PT-SH | PT-SH1 | 100 +100 | 30,548,578 | 3.05 | 46 | Q40 |
|  | PT-SH2 | 100 +100 | 27,786,467 | 2.78 | 46 | Q39 |
|  | PT-SH3 | 100 +100 | 34,759,047 | 3.47 | 47 | Q39 |
| Total |  |  | 93,094,092 | 9.30 |  |  |

**Table S3** Statistics of transcriptome assembly

| Type | Assembled unigenes | Assembled transcripts |
| --- | --- | --- |
| Total sequence number | 260,372 | 652,203 |
| Average length (bp) | 514 | 697 |
| Smallest length (bp) | 201 | 201 |
| Largest length (bp) | 14,092 | 15,057 |
| N50 length (bp) | 620 | 944 |

**Table S4** The summary of Differentially Expresed Genes (DEGs)

| Comparison | DEGs | Up-regulated | Down-regulated |
| --- | --- | --- | --- |
| OC vs NOC | 3,028 | 2,584 | 444 |
| PT vs NOC | 19,895 | 9,973 | 9,922 |
| PT-SH vs NOC | 27,692 | 13,896 | 13,796 |
| PT vs OC | 314 | 222 | 92 |
| PT-SH vs OC | 1,521 | 956 | 565 |
| PT-SH vs PT | 885 | 525 | 360 |

**Table S5** The summary of GO enrichment of the differentially expressed genes

| Combination | | OC vs NOC | PT vs OC | PT-SH vs PT |
| --- | --- | --- | --- | --- |
| GO enrichment | |  |  |  |
| BP | # GO terms | 62 | 39 | 112 |
|  | # GO significant unigenes | 1,851 | 462 | 7,778 |
|  | # up-regulated unigenes | 1,743 (94.2%) | 375 (81.2%) | 4,886 (63.6%) |
|  | # down-regulated unigenes | 108 | 87 | 2,799 |
| MF | # GO terms | 79 | 16 | 19 |
|  | # GO significant unigenes | 2,367 | 180 | 226 |
|  | # up-regulated unigenes | 2,209 (93.3%) | 93 (51.7%) | 124 (54.9%) |
|  | # down-regulated unigenes | 158 | 87 | 102 |
| CC | # GO terms | 42 | 8 | 12 |
|  | # GO significant unigenes | 7,971 | 206 | 328 |
|  | # up-regulated unigenes | 7,345 (92,2%) | 112 (54.4%) | 119 (36.3%) |
|  | # down-regulated unigenes | 626 | 94 | 209 |

**Table S6** Top significant enriched GO terms amongst the differentially expressed genes*

| Comparison | OC vs NOC | | PT vs OC | | PT-SH vs PT | |
| --- | --- | --- | --- | --- | --- | --- |
| Gene Ontology | GO term | # significant / annoted unigenes (Fisher test: FDR) | GO term | # significant / annoted unigenes (Fisher test: FDR) | GO term | # significant / annoted unigenes (Fisher test: FDR) |
| BP | photosynthesis  (GO:0015979) | 120 /587  (2.4e-17) | carbohydrate metabolic process (GO:0005975) | 41/3006  (1.4e-10) | transcription, DNA-templated (GO:0006351) | 138/6501  (1.4e-0.6) |
|  | oxylipin biosynthetic process (GO:0031408) | 26/89  (4.6e-15) | plant-type primary cell wall biogenesis (GO:0009833) | 9/117  (3.7e-09) | water transport  (GO:0006833) | 10/47  (3.3e-10) |
|  | secondary metabolite biosynthetic process (GO:0044550) | 56/380  (1.4e-15) | cell wall organization (GO:0071555) | 19/1224  (3.5e-0.6) | response to abscisic acid  (GO:0009737) | 49/1453  (9.1e-0.7) |
|  | oxidation-reduction process (GO:0055114) | 158/1813  (7.3e-13) | basic amino acid transport (GO:0015802) | 3/14  (3.3e-0.5) | xyloglucan metabolic process (GO:0010411) | 11/96  (4.0e-0.8) |
|  | lignin biosynthetic process (GO:0009809) | 23/99  (8.2e-08) | multidimensional cell growth (GO:0009825) | 5/108  (0.00014) | auxin catabolic process (GO:0009852) | 4/8  (1.7e-06) |
|  | response to oxidative stress (GO:0006979) | 77/922  (4.1e-08) | auxin catabolic process (GO:0009852) | 2/5  (0.00020) | hydrogen peroxide catabolic process (GO:0042744) | 11/144  ( 2.5e-06) |
|  | plant-type primary cell wall biogenesis (GO:0009833) | 21/119  (4.3e-08) |  |  | urea transport (GO:0015840) | 5/11  (1.4e-07) |
| MF | heme binding  (GO:0020037) | 77/659  (2.2e-15) | beta-galactosidase activity (GO:0004565) | 13/72  (2.4e-17) | DNA binding transcription factor activity (GO:0003700) | 84/2850  (2.5e-13) |
|  | pigment binding  (GO:0031409) | 12/25  (1.2e-10) | cellulose synthase activity (GO:0016759) | 10/142  (1.8e-09) | water channel activity (GO:0015250) | 12/45  (2.8e-13) |
|  | iron ion binding  (GO:0005506) | 64/627  (1.8e-10) | UDP-glucosyltransferase activity (GO:0035251 | 13/566  (3.8e-06) | xyloglucan:xyloglucosyl transferase activity (GO:0016762) | 11/56  (1.0e-10) |
|  | oxidoreductase activity, with incorporation or reduction of molecular oxygen, NAD(P)H (GO:0016709) | 36/266  (1.2e-09) | carbohydrate binding (GO:0030246) | 16/936  (1.2e-05) | alcohol dehydrogenase (NAD) activity (GO:0004022) | 7/31  (9.9e-08) |
|  | glyceraldehyde-3-phosphate dehydrogenase (NADP+) activity (GO:0047100) | 8/9  (9.8e-11) | xyloglucan 1,6-alpha-xylosidase activity (GO:0080176) | 2/6  (0.00034) | heme binding (GO:0020037) | 26/640  (2.3e-07) |
|  | linoleate 13S-lipoxygenase activity (GO:0016165) | 10/21  (4.7e-09) | alpha-D-xyloside xylohydrolase (GO:0061634) | 2/7  (0.00047) | structural constituent of cell wall (GO:0005199) | 6/25  (5.7e-07) |
|  | acylglycerol lipase activity (GO:0047372) | 10/29  (1.9e-07) | amino acid transmembrane transporter activity (GO:0015171) | 5/137  (0.00053) | iron ion binding (GO:0005506) | 22/611  (1.3e-05) |
| CC | chloroplast thylakoid (GO:0009534) | 157/911  (< 1e-30) | apoplast  (GO:0048046) | 16/585  (3.8e-09) | central vacuole  (GO:0042807) | 9/15  (3.1e-14) |
|  | plastoglobule  (GO:0010287) | 26/116  (2.1e-12) | plant-type cell wall (GO:0009505) | 11/415  (1.6e-06) | primary cell wall  (GO:0009530) | 5/6  (1.7e-09) |
|  | chloroplast envelope (GO:0009941) | 117/1,451  (1.7e-11) | anchored component of membrane (GO:0031225) | 8/334  (8.7e-05) | integral component of plasma membrane  (GO:0005887) | 27/743  (9.3e-07) |
|  | photosystem II oxygen evolving complex (GO:0009654) | 21/36  (3.6e-20) | cellulose synthase complex (GO:0010330) | 3/36  (0.00046) | apoplast  (GO:0048046) | 22/593  (6.9e-06) |
|  | photosystem I reaction center (GO:0009538) | 9/11  (2.1e-11) |  |  |  |  |
|  | integral component of membrane (GO:0016021) | 612/11,547  (9.0e-12) |  |  |  |  |
|  | apoplast  ( GO:0048046) | 61/622  (1.2e-09) |  |  |  |  |
| *Complete lists of significant enriched GO terms of DEGs in the biological process, molecular function and cellular compartment categories are enclosed as Suppl. data S3, S4 and S5, respectively. | | | | | | |
