# Supplementary figures and images for: Protuberances are organized distinct regions of long-term callus: histological and transcriptomic analyses in kiwifruit
Source: Plant Cell Rep. 2021 Feb 5;40(4):637–65. doi: 10.1007/s00299-021-02661-0 (PMC7954764; doi:10.1007/s00299-021-02661-0)

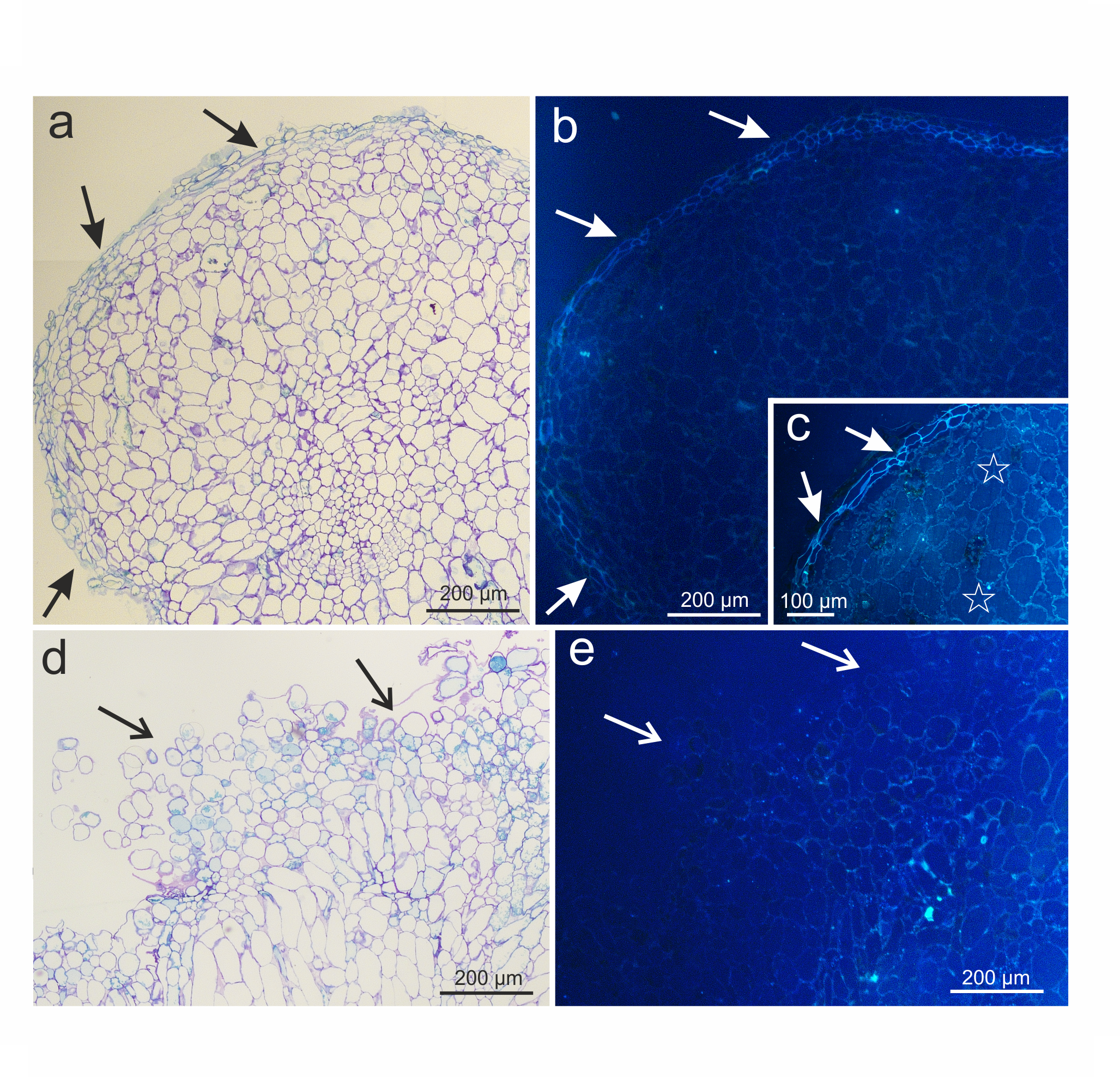

Supplement: Supplementary file 2 — Supplementary file2 Suppl. Fig. 1 Longitudinal sections of detached PT (a–c) and OC (d, e) in Actinidia chinensis cv. deliciosa; TBO (a, d) and auramine O (b, c, e) staining. a The part of ball-shaped PT with the compact composition of cells (black arrows) on the surface. b White arrows indicate cells on the surface where the cell walls show fluorescence (b, c); notice the autofluorescence of cell walls (stars). d, e OC with the surface composed of loosely attached cells (black open arrows) which show autofluorescence (white open arrows) (TIF 7556 KB) [file 299_2021_2661_MOESM2_ESM.tif]

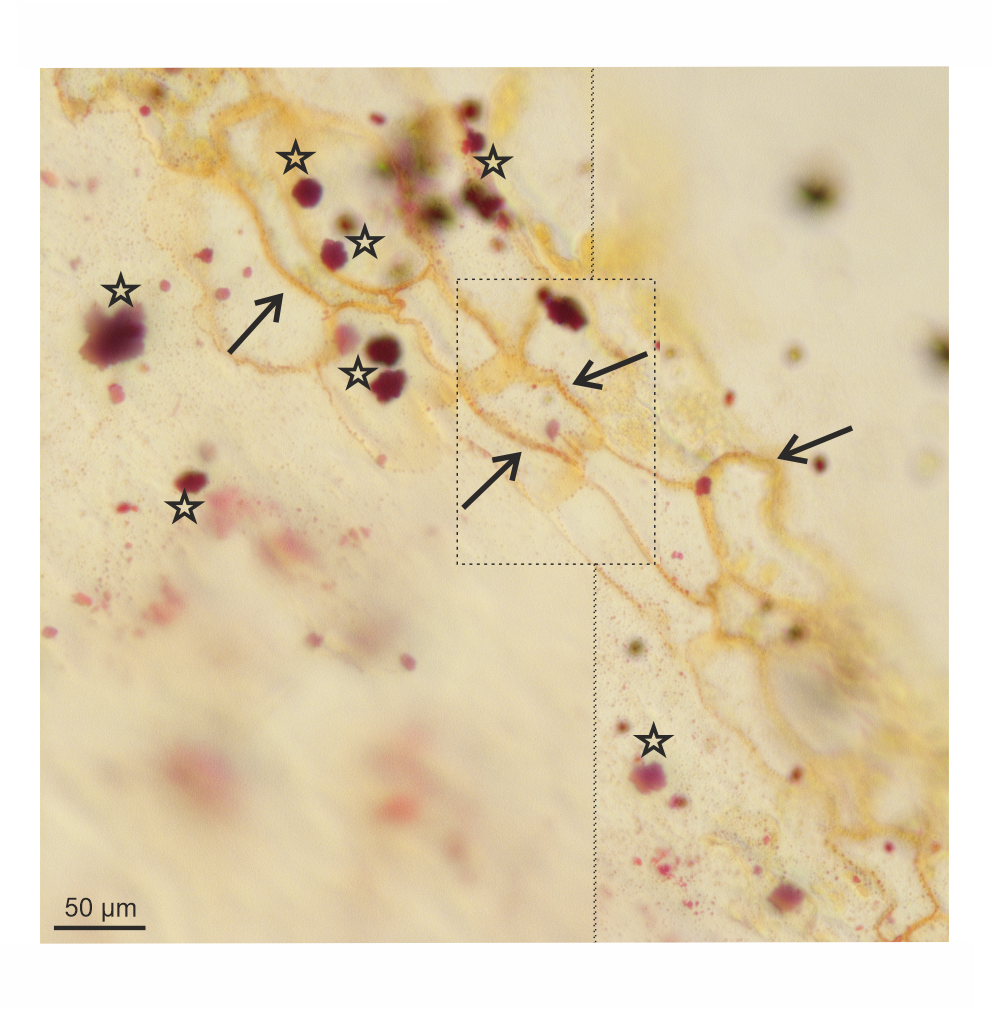

Supplement: Supplementary file 3 — Supplementary file3 Suppl. Fig. 2 Magnification of longitudinal sections of detached PT in Actinidia chinensis cv. deliciosa; Sudan III staining. Arrows indicate cells on the surface with a reddish color of the cell walls. Stars show the crystals of dye in the background. Dotted lines indicate lines of the merged pictures (TIF 1399 KB) [file 299_2021_2661_MOESM3_ESM.tif]
